# Organ-on-a-Chip and Microfluidic Plant Cell Culture Systems: The Next Frontier for Controlled Secondary Metabolite Production and Real-Time Metabolomic Monitoring

**DOI:** 10.3390/plants15142179

**Published:** 2026-07-16

**Authors:** Abhishek Dadhich, Vikas Sharma, Iyyakkannu Sivanesan

**Affiliations:** 1 Department of Biotechnology, Graphic Era (Deemed to be University), Dehradun 248002, Uttarakhand, India; sabhi5061@gmail.com; 2 School of Bioengineering and Biosciences, Lovely Professional University, Jalandhar 144411, Punjab, India; biotech_vikas@rediffmail.com; 3 Department of Environmental Health Science, Human and Eco Care Center, Konkuk University, Hwayang-dong, Gwangjin-gu, Seoul 05029, Republic of Korea

**Keywords:** plant microfluidics, secondary metabolites, real-time metabolomics, elicitation, synthetic biology, metabolic engineering

## Abstract

Plant secondary metabolites remain indispensable for pharmaceuticals, nutraceuticals, and cosmeceuticals, yet conventional plant culture systems are increasingly limited by inconsistent yields, poor scalability, and inadequate capacity for real-time process monitoring. Microfluidic technologies and organ-on-a-chip (OoC) platforms, originally developed for mammalian biology, are now emerging as powerful tools to overcome these constraints. These systems enable laminar flow, precise gradient generation, single-cell resolution, and biosensor integration, providing unprecedented control over the cellular microenvironment and supporting non-destructive, real-time metabolomic monitoring. While recent reviews have surveyed plant microfluidics broadly covering developmental biology, single-cell phenotyping, and root–microbe interactions, this review provides, to our knowledge, the first synthesis focused specifically on organ-on-a-chip approaches for plant secondary metabolite biosynthesis and real-time metabolomic monitoring. Advances in device fabrication, including PDMS, paper-based, hydrogel, and thermoplastic materials, surface engineering, gradient-based elicitation strategies, and integration of optical, electrochemical, and mass spectrometric detection systems have also been critically examined. Special emphasis is placed on root-on-a-chip, shoot meristem, protoplast, callus, and 3D organoid platforms for studying cell wall mechanics, vacuolar dynamics, cytoskeletal responses, and signalling cascades. However, challenges remain in long-term culture stability and scalability; nonetheless, these technologies offer a roadmap toward programmable ‘plant biosynthetic factories’ to produce high-value natural products.

## 1. Introduction

The market for plant-based pharmaceuticals, nutraceuticals, and cosmeceuticals is expanding rapidly worldwide, with an estimated annual value exceeding USD 150 billion [1]. The rise in consumer demand for natural products and the complexity of plant secondary metabolites in terms of structure, which is unmatched in total chemical synthesis, are driving the growth. Among commercially marketed plant-derived pharmaceuticals, nutraceuticals, and high-value phytochemicals, more than 80% are reported to remain dependent on extraction from whole plants or on biological (cell/tissue culture) production systems, rather than total chemical synthesis, owing to structural complexity [2,3]. These include terpenoids such as artemisinin and paclitaxel precursors, alkaloids such as vinblastine, and phenylpropanoids such as resveratrol [2,3]. However, traditional sourcing from wild or cultivated plants is plagued by chronic problems, including seasonality, slow growth, environmental issues, overharvesting of endangered plants, and supply chain disruptions. In response, plant cell and tissue culture technologies have been developed as alternatives since the mid-20th century. These platforms provide controlled environments for biomass propagation and metabolite accumulation [4].

Although innovative optimization efforts have been implemented over the years, conventional plant cell suspension cultures, hairy root cultures, and bioreactor systems are still plagued by frequent limitations [5]. These limitations include low and variable secondary metabolite (SM) yields due to dedifferentiation and loss of tissue-specific biosynthetic capacity. Additional problems are shear sensitivity in large-scale stirred-tank bioreactors and long lag times. Critically, conventional systems cannot mimic the spatiotemporal gradients of nutrients, elicitors, and signalling molecules that regulate metabolite biosynthesis in intact plants [6,7]. Flask-scale experiments have demonstrated significant improvements with elicitation treatments such as jasmonates, salicylic acid, and pathogen-associated molecular patterns (PAMPs), but transferring these responses to an industrial level can lead to less consistent responses, cell variability, and limited process analytical technology (PAT) to monitor the process in real time [8]. Many high-value compounds remain limited in titer for cost-effective commercialization, indicating the need for disruptive platforms that integrate microscale precision with dynamic control of environmental conditions [9].

**Figure 1:**
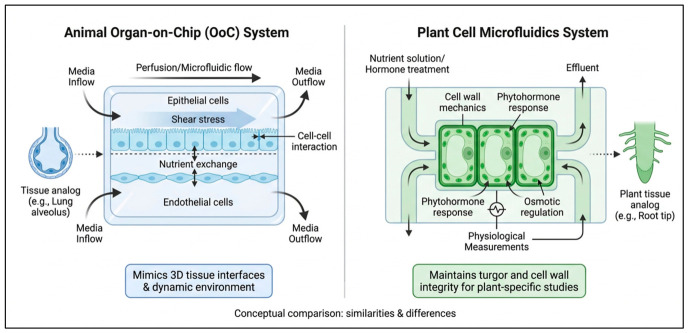
Conceptual comparison of animal organ-on-a-chip (OoC) systems and plant cell microfluidic platforms [12,18,19].

An appealing approach to these issues is microfluidic technologies and organ-on-a-chip (OoC) platforms, which were first developed around mammalian cell biology [10]. OoC systems have transformed biomedical research since the discovery of a breathing lung-on-a-chip by [11,12], which recreated tissue interfaces, mechanical cues, and a vascular-like perfusion in micrometer-scale channels. Such devices take advantage of laminar flow patterns, fine control of the gradient and integration of biosensors to provide a physiologically relevant microenvironment that surpasses static culture in simulating in vivo conditions [13]. Conceptual translation to plant systems is logical and timely. Plant vascular tissues, such as xylem and phloem, are naturally analogous to the perfused channels of animal OoC devices. Similarly, plant cell wall mechanics, turgor pressure, vacuolar compartmentation, and local defence signalling correspond to microfluidic confinement, surface functionalization, and spatiotemporal stimulus delivery. Recent microfluidic research has mainly centered on developmental processes such as root growth, pollen tube guidance, and single-cell analysis, which have provided important building blocks but have not been extensively studied in the context of secondary metabolism or real-time metabolomics [14] (Figure 1).

This has been addressed with recent advances. Suspension cell and rhizosphere-on-a-chip models have shown improved cell viability and increased responsiveness to elicitors, while shoot meristem cultures and 3D callus or organoid-like structures have shown the ability to deliver chemical gradients on-chip, with improved cell viability and increased elicitor responsiveness. Examples include modular bioreactors for plant cell–cell communication studies, paper-based devices, polydimethylsiloxane (PDMS)-based devices for precise application of abiotic and biotic stresses, and integrated systems that combine perfusion with optical/electrochemical detection [15]. However, the available literature is highly fragmented, does not provide a synthesis of engineering aspects or specific biological phenomena, and only a few papers address the synthesis of controlled SM production. While most studies have demonstrated successful results in model systems such as *Arabidopsis*, *Taxus*, *Catharanthus*, or *Vitis*, comprehensive evaluation of yield benchmarks relative to conventional bioreactors, long-term culture stability, and the integration of advanced analytics is still emerging [16,17].

Several recent reviews have addressed adjacent aspects of plant microfluidics. Marczakiewicz-Perera et al. [18] and Sanati Nezhad [19] provide broad engineering-oriented surveys of microfluidic platform design for plant physiology and development, with limited treatment of secondary metabolism or analytical integration. Pradhan et al. [14] focus on single-cell technologies for plant biotechnology generally, spanning genotyping and phenotyping applications beyond metabolite biosynthesis. Marín-Lizarraga et al. [20] and Zhao et al. [21] catalogue microfluidic chip architectures for plant systems from single cells to whole plants, emphasizing device taxonomy rather than biosynthetic output or metabolomic analytics.

Building on this identified gap, the present review offers, to our knowledge, the first narrative review of organ-on-a-chip design principles specifically applied to precision biotransformation of secondary metabolites and on-line metabolomics monitoring in plant physiological systems. Taking an approach somewhat like that adopted by the Science in Microgravity Science Gateway, we consider only literature published since about 2005 that describes true microfluidic environments (usually with channels and chambers less than 1 mm) containing living plant cells or tissues, excluding macroscale or millifluidic adaptations. Our synthesis is novel in several ways: (1) it explicitly brings together the advantages of animal OoC methods and plant-specific challenges like rigid cell walls, high turgor, photosynthetic needs, and vacuolar storage; (2) it critically evaluates the unique advantages of microfluidics for spatiotemporal elicitation and non-destructive, high resolution metabolomics, which is not provided by bulk cultures that average over time and space; (3) it combines perspectives from plant biology, microfluidic engineering, synthetic biology, and computational modelling to outline a trajectory towards programmable ‘plant biosynthetic factories’. The synthesis also seeks to develop new platforms that more effectively connect animal OoC methodologies to plant systems, thereby fostering cross-disciplinary innovation and advancing the development of novel platforms for sustainable, high-yield, on-demand production of plant-derived bioactives.

Despite growing interest in plant microfluidics, a clear gap remains: no existing work brings together secondary metabolite production and real-time metabolomic monitoring under one organ-on-a-chip framework; the literature remains scattered across device engineering, developmental biology, and single-cell phenotyping papers separately. This review aim to close that gap with three objective: (1) critically examine device fabrication, materials, surface chemistry, and sensor integration developed for plant cell microfluidics; (2) analyze how microfluidic confinement and spatiotemporal elicitation control cell wall mechanics, vacuolar dynamics, cytoskeletal response, and signalling cascade that govern secondary metabolite biosynthesis; and (3) evaluate emerging real-time metabolomic detection platforms alongside their integration with synthetic biology, CRISPR screening, and computational/digital-twin modelling toward programmable plant biosynthetic factories. Scope limited to literature since ~2005 describing true microfluidic environments (channel/chamber < 1 mm) containing living plant cells or tissue; macroscale/millifluidic system, general animal OoC engineering, and conventional bioreactor optimization fall outside scope and the reader is directed to existing reviews on these topics [14,18,19,20,21]. Through this focused synthesis, this review aims to provide a roadmap connecting engineering, plant physiology, analytical chemistry, and synthetic biology perspectives toward a next-generation, sensor-integrated plant bioprocessing platform.

## 2. Microfluidic Platform Design and Functional Components for Plant Cell Analysis

Conventional methods of plant cell analysis, relying on growth in soil pots or agarose plates and subsequent phenotypic screening in traditional greenhouses, are costly, suffer from low spatial resolution, and lack the ability to manipulate plant cells locally and accurately. To overcome these limitations, microfluidic platforms have become powerful tools in plant cell biology [19]. From single-cell trapping devices to droplet-based screening systems to organ-on-a-chip and root–microbe interaction modules, microfluidic platforms provide unprecedented control over microscale environments for the cultivation, manipulation, and analysis of plant cells, tissues, and organs. Novel devices have been developed to analyze plant cells at micrometer-scale spatial resolution, which mimics the internal microenvironment of plant cells and allows the study of gene expression [22] and cell biomechanics, growth mechanisms, cell division, and cell fusion [23]. Microfluidic chips are usually designed using AutoCAD software and fabricated by soft photolithography, after which PDMS is cast, cured, and permanently bonded to a glass surface by oxygen plasma treatment, thereby providing optical transparency and biocompatibility for live-cell imaging. These chips feature functional units containing a series of pillars with gaps between them (measured in micrometers) and V-shaped cups to physically separate each cell, along with pneumatic membrane valves controlled by external pressure sources to precisely regulate fluid flow [18,24]. Complementing these PDMS-based architectures, a broader spectrum of microchamber configurations, including pentagonal protoplast cultivation arrays, dual-layer metabolite exchange chips, and large-scale oval root chambers, has been validated across plant species ranging from tobacco protoplasts to whole Cannabis sativa root systems, demonstrating that chamber geometry must be tailored to the specific biological scale of the target tissue.

Moreover, by combining microscale electrodes with structured microfluidic channels, non-invasive impedance platforms have been developed to quantify the status of single plant cells, such as primary cell wall regeneration, a process that involves the complex assembly and deposition of cellular components. Together, these platforms illustrate high-throughput cellular analysis with increased experimental accuracy, reduced costs and time, design flexibility, the ability to perform large-scale combinatorial screening, and the integration of miniaturized sensors, making microfluidic technology an increasingly important cornerstone methodology in contemporary plant cell biology (Table 1).

### 2.1. Microfluidic Fabrication: PDMS, Paper, and Emerging Materials

The substrate material on which the microfluidic device is fabricated significantly influences the device’s mechanical and chemical strength, as well as its potential for translation in biological research [25]. The choice of PDMS as the substrate for microfluidic device fabrication has endured since the early days of soft lithography in the late 1990s, owing to its optical transparency, gas permeability, ease of prototyping via replica molding, and inherent biocompatibility [26]. In this process, a photolithography master mould is cast with elastomer and plasma-bonded to glass. This allows rapid fabrication of devices with sub-micrometer feature resolution in just 24–48 h, enabling widespread adoption in cell biology and biomechanics laboratories [27]. However, the dominance of PDMS has been questioned in recent years due to its physicochemical limitations at interfaces with complex biological systems [28]. One of the many perils of PDMS is its ability to absorb small hydrophobic molecules, including hormones, fluorescent dyes, signalling lipids, and pharmaceutical compounds, in both pharmacology and plant hormone biology [25,26]. Hermann et al. (2025) [29] showed that estradiol and other steroidal compounds were rapidly removed from culture medium upon contact with PDMS surfaces. This finding casts doubt on the validity of hormone perturbation studies conducted in standard soft-lithography chips. Comparative studies have shown that small-molecule uptake in PDMS channels can be orders of magnitude faster than in glass or thermoplastic counterparts. This has prompted the community to explore surface passivation techniques, including sol–gel coating, parylene-C deposition, and PEGylation. Each approach offers partial mitigation but adds fabrication complexity [30]. To address these limitations, paper-based microfluidics was developed as a significantly cheaper alternative, particularly for point-of-care diagnostics and in resource-limited environments [31]. The Whitesides group pursued the idea of exploiting capillary-driven flow in hydrophilic cellulose matrices patterned with hydrophobic barriers created by wax printing, photolithography, or inkjet deposition, which he dubbed microfluidic paper analytical devices (µPADs). The low cost of the paper substrate, its biodegradability, and its compatibility with colorimetric readout have made µPADs appealing for lateral flow immunoassays and heavy metal detection in agricultural water [32,33]. However, due to the stochastic architecture of fibers, they exhibit inherent variability in flow velocity and sample dispersion, which limits their application in precision quantitative biology, especially in achieving subcellular spatial resolution and controlled laminar gradients. These limitations have been partially overcome, and the analytical throughput of a paper-based platform has increased with recent progress in 3D µPADs (structured paper-based analytical devices), made possible by stacking and folding paper layers with programmed fluid routing [34].

Beyond PDMS and paper-based systems, several emerging fabrication approaches are redefining biocompatibility standards for plant cell microfluidics. Thiol-ene and off-stoichiometry thiol-ene (OSTE) polymers have emerged as alternatives to PDMS. They offer near-zero absorption of small molecules and direct surface functionalizability without plasma treatment. These properties are especially important for plant hormone studies, where PDMS-mediated hormone depletion, as demonstrated for estradiol by Hermann et al. (2025), fundamentally undermines experimental validity [31]. Cyclic olefin copolymer (COC), already summarised in Table 1, exhibits orders-of-magnitude lower absorption of jasmonates and salicylic acid than PDMS, making it the preferred substrate for elicitor-based secondary metabolite studies where concentration fidelity across the chip must be maintained [27]. Additive manufacturing approaches, particularly stereolithography (SLA) and two-photon polymerization (2PP), extend fabrication capabilities to complex three-dimensional geometries, including branching channel networks that mimic xylem architecture and sub-micron constrictions suitable for single protoplast trapping, though mandatory post-cure passivation to address resin cytotoxicity remains a prerequisite for biological deployment [24]. Most relevant to plant systems, bioprinted cellulose nanofiber composite channels present a chemically authentic surface. They mimic the β-1,4-glucan backbone of the primary cell wall, reducing non-specific polysaccharide adsorption. They also provide mechanically tunable scaffolds within the stiffness range relevant to callus and organoid culture [33].

### 2.2. Flow Regimes, Gradient Generation, and Mass Transport

Precise manipulation of fluid flow and chemical microenvironments lies at the heart of microfluidic biology. An understanding of the governing transport physics is essential for interpreting experimental outcomes in these systems. At the microscale, fluid dynamics are characterized by the dominance of viscous over inertial forces, formally expressed through the dimensionless Reynolds number Re = ρvL/μ, which in typical biological microfluidic channels (channel widths of 10–500 μm, flow velocities of 10 μm s^−1^ to 10 mm s^−1^) invariably falls well below unity, situating flows firmly in the laminar regime [35,36]. The absence of turbulent mixing was initially seen as a limitation. However, it has since been exploited to generate stable, reproducible concentration gradients through co-laminar juxtaposition of adjacent streams. This capability underpins a large body of research on chemotaxis, morphogen gradients, and root tropism.

The seminal contribution of Dertinger et al. (2001) established the Christmas-tree gradient generator as the canonical architecture for linear concentration gradients, wherein serial dilution through symmetric bifurcating networks produces multiple streams of defined composition that merge into a single channel [37]. This design has since been adapted into nonlinear, temporal, and combinatorial gradient formats by numerous groups. Bhattacharjee and Bhattacharya (2019) demonstrated a reconfigurable gradient generator capable of switching between monotonic and bell-shaped profiles on timescales of seconds, enabling the study of plant root chemotropism in dynamic soil chemical environments that more faithfully replicate rhizospheric conditions than static gradient chambers [38]. Nevertheless, a critical limitation acknowledged throughout this literature is the sensitivity of gradient stability to fluctuations in external pressure and to the compliance of PDMS channel walls under varying flow rates, a concern that has motivated the adoption of on-chip pneumatic flow stabilizers and rigid COC substrates in precision gradient studies [39,40].

Beyond advection-dominated flow, diffusion governs molecular transport on length scales below the Péclet number transitional regime relevant to the transport of large signalling molecules and particles in narrow channels or stagnant zones. The Péclet number Pe = vL/D, where D is the molecular diffusivity, delineates whether convective or diffusive processes dominate. For small molecules such as auxin (D ≈ 6 × 10^−10^ m^2^ s^−1^) at typical chip flow velocities, Pe values near unity indicate that both mechanisms contribute comparably to concentration profiles at channel junctions [35]. This has practical consequences for gradient fidelity. Diffusive blurring progressively erodes sharp gradients at longer channel lengths and lower flow rates, necessitating careful optimization of channel geometry for applications that require steep spatial concentration steps, such as studies of differential auxin distribution during root gravitropic responses. PDMS absorption of small hydrophobic molecules, including phytohormones, can distort gradient profiles in plant microfluidic channels, as modelled by Grant et al. (2021) for organ-chip drug concentrations [41]. mandating surface treatment protocols Taken together, the physics of microscale flow and transport do not merely constrain device design; they constitute an active experimental parameter that, when understood and controlled, enables the recapitulation of biologically meaningful chemical microenvironments with unprecedented spatial and temporal resolution.

### 2.3. Surface Chemistry and Biocompatibility for Plant Cells

An important and often overlooked parameter in the fidelity of experiments in microfluidic biology is the interface formed between the walls of the microchannel and the biological materials studied; this becomes highly significant when the biological materials are plant cells, which possess complex cell walls made up of biopolymer-based materials, chemically active surfaces, and mechanical sensitivity requiring a high level of compatibility with protocols developed for mammalian cell culture. In animal cell culture systems, rapid conditioning of the non-specific native PDMS surfaces by serum proteins leads to hydrophobic, protein-adsorbing surfaces and surface chemistry; in plant protoplasts and whole cells loaded into microchannels, the non-specific adsorption of proteins, including signalling peptides and polysaccharide wall fragments, as well as applied hormone stimuli, can complicate the chemical environment being studied [42,43,44].

In the last decade, strategies for surface functionalization have evolved significantly for plant cell microfluidic systems; while based on a broader range of surface chemistry tools, they still need to be optimized for the plant system. Oxygen plasma treatment is the most widely used method to make PDMS hydrophilic for bonding; it creates transient surface silanolisation that restores a hydrophobic state in a matter of hours because of chain recovery, a kinetic instability that hinders stable contact with aqueous solutions, and which is the reason for the development of alternative protocols for surface modification that are more durable [45,46]. Of these, covalent grafting of polyethylene glycol (PEG) brushes via silane chemistry has become a dominant technique, yielding surfaces that are resistant to non-specific adsorption of proteins and polysaccharides and are optically clear, suitable for fluorescence microscopy [47,48]. Poly-L-lysine and polylysine-PEG have also been used to provide a positively charged surface, suitable for the net negative charge of plant cell walls, to gently and non-damagingly attach plant roots to the channel surface for live-cell confocal imaging of meristematic cells [41,49].

All of these studies showed that PDMS chips with PEG-treated walls and controlled atmosphere composition could support root elongation rates and meristematic cell division patterns within 24–72 h windows of observation that were indistinguishable from the rates and patterns observed by agar control plants, a critical validation that made microfluidics a viable platform for plant biology in a way that was technically interesting but also biologically credible. More recently, the introduction of agarose gel-filled microchannels, which mimic the mechanical compliance of the soil matrix and deliver defined chemical gradients, has added another dimension of physiological relevance, allowing studies of root gravitropism and hydrotropism under mechanically realistic conditions [49]. All these advances demonstrate that a single material solution will not permit true biocompatibility of plant cells; it is a multi-parameter optimization problem that remains a challenge for materials innovation in this field.

A critical gap that compounds these material-level challenges is the absence of a standardized biocompatibility validation framework for plant microfluidic devices, an analog to ISO 10993 for biomedical implants that does not yet exist in the plant science community. Without consensus validation criteria, biocompatibility claims across studies remain difficult to compare or reproduce. A tiered validation approach is therefore proposed: beginning with cytotoxicity screening using plant-specific viability markers such as Evans Blue exclusion and fluorescein diacetate/propidium iodide fluorescence, progressing to transcriptomic comparison of chip-cultivated versus agar-grown cells as demonstrated by the Bhalerao laboratory [50], and culminating in full metabolomic fingerprinting to confirm that biosynthetic pathway integrity is not silently perturbed by device material interactions. On the surface chemistry front, zwitterionic polymer coatings, specifically carboxybetaine and sulfobetaine silane derivatives, represent the most promising next-generation treatment for plant microfluidic channels, as they simultaneously resist protein and polysaccharide adsorption, remain stable beyond seven days under perfusion conditions, and critically do not sterically exclude small hormonal ligands in the way that PEG brushes can, preserving the chemical authenticity of the microenvironment [43]. Complementing chemical surface modification, laser-etched nano-topographic micropatterning on COC and glass substrates offers a purely physical strategy to orient plant cell growth along defined axes without chemical intervention, an approach particularly valuable for root tip imaging chips, where meristematic cell file organization must be preserved without introducing surface chemistry confounds [40].

### 2.4. Integration of Sensors: Optical, Electrochemical, and Acoustic

The analytical power of a microfluidic platform is ultimately realized through sensors that transduce biological and chemical information from the chip into measurable signals. The integration of sensing modalities directly within microfluidic architectures, rather than relying on external analytical instruments, represents one of the most consequential ongoing engineering challenges. Several imperatives drive on-chip sensor integration. First, miniaturization reduces sample volume requirements to the sub-nanolitre range, enabling non-destructive monitoring of single cells. Second, spatial co-location of stimulus delivery and sensing eliminates temporal and spatial offsets that confound sequential measurements. Third, multiplexed sensor arrays enable simultaneous acquisition of orthogonal physical and chemical parameters, thereby constructing multidimensional biological response landscapes [51,52].

Optical sensing modalities are the most widely adopted approach, capitalizing on the inherent optical transparency of glass- and PDMS-based microfluidic chips and the maturity of fluorescence microscopy. Laser-induced fluorescence (LIF) detection integrated within microfluidic channels has achieved sub-nanomolar detection limits for fluorescently labelled metabolites, enabling real-time monitoring of reactive oxygen species (ROS) production in response to pathogen elicitors in plant root cells, a measurement previously accessible only through destructive biochemical assays [53,54]. The coupling of microfluidics with confocal and light-sheet fluorescence microscopy has opened the ability to acquire four-dimensional (3D + time) imaging data on living plant cells experiencing precisely defined chemical perturbations, as exemplified by studies tracking PIN-mediated auxin efflux carrier relocalization during gravitropic bending in real time within chip-cultivated Arabidopsis roots [55]. However, the requirement for high numerical aperture objectives constrains chip geometry to thin-profile designs with working distances below 300 μm, and the phototoxicity associated with prolonged fluorescence excitation demands careful optimization of imaging duty cycles in longitudinal experiments.

Electrochemical sensors integrated within microfluidic chips offer complementary capabilities, particularly for the label-free, real-time detection of redox-active species and ionic analytes that cannot be readily tagged with fluorescent probes. Amperometric microelectrodes fabricated from gold, platinum, or carbon nanotube-modified surfaces have been integrated into microchannel walls to detect hydrogen peroxide, nitric oxide, and phytohormones with picomolar sensitivity [56,57]. Amperometric microneedle sensors have demonstrated simultaneous in vivo detection of IAA and salicylic acid from living plant tissue with µM sensitivity [58]. Ion-selective electrode arrays embedded in polycarbonate chips have further enabled real-time measurement of K^+^, Ca^2+^, and H^+^ effluxes from individual root zones, data previously accessible only through non-invasive microelectrode techniques (MIFE) performed under stringent mechanical isolation conditions [59,60]. The electrochemical integration approach is not without limitations: electrode fouling by polysaccharide exudates and phenolic compounds from plant cells degrades sensor performance over multi-hour experiments, necessitating periodic regeneration or the use of antifouling electrode coatings such as Nafion or conducting polymer films.

Acoustic sensing, though comparatively nascent in its integration with biological microfluidics, has emerged as a powerful label-free modality for quantifying cell mass, viscoelasticity, and adhesion dynamics. Surface acoustic wave resonators and suspended microchannel resonators (SMRs) embedded in microfluidic chips can detect mass changes at the femtogram scale, enabling the measurement of cell buoyant mass accumulation rates as a proxy for growth, a parameter of direct relevance to studies of plant cell expansion and growth in response to hormonal or mechanical stimuli [61,62]. Building on the pioneering SMR work of the Manalis laboratory [63], subsequent adaptations of the resonator geometry to accommodate the larger dimensions of plant protoplasts and root cells have demonstrated that acoustic mass sensing can resolve growth rate differences of less than 1% per hour in single plant cells, a resolution impossible to achieve with optical volumetry at comparable throughputs. Acoustic radiation force, generated by bulk acoustic wave (BAW) transducers integrated beneath microfluidic channels, has additionally been applied to the contactless manipulation and positioning of plant cells within defined chip regions, enabling the formation of defined multi-cell aggregates for the study of cell-to-cell signalling without the chemical perturbation associated with physical contact-based manipulation [64].

The convergence of these sensing modalities with closed-loop process control represents a pivotal step toward addressing one of the central limitations of conventional plant cell bioreactors, as identified in the Introduction: the lack of adequate process analytical technology (PAT). In microfluidic systems, PAT integration becomes tractable through multiplexed electrochemical sensor arrays that simultaneously monitor pH, dissolved oxygen, and target metabolite concentrations from a single chip, a capability that is essential because plant secondary metabolism is acutely sensitive to microenvironmental fluctuations that, at larger culture volumes, propagate in spatially heterogeneous and poorly predictable ways [60]. Impedance spectroscopy offers a complementary, non-invasive modality for real-time assessment of cell density and viability, particularly valuable in dense, optically opaque three-dimensional callus cultures where fluorescence-based methods fail and whose electrode-based architecture scales naturally to multi-well and multi-module formats without the optical access constraints that limit confocal approaches [53]. For metabolite quantification specifically, inline Raman spectroscopy with fiber-optic probes has emerged as the most promising label-free tool at pilot scale, delivering continuous molecular fingerprints without sampling or extraction; the principal bottleneck remains incomplete spectral libraries for plant secondary metabolites, though machine learning-assisted deconvolution is progressively narrowing this gap as discussed further in Section 6.2 [52]. Critically, when each chip module within a scaled-out array transmits real-time sensor data to a centralized process controller, autonomous per-module optimization of flow rate, elicitor concentration, and nutrient composition becomes achievable realising the numbering-up strategy that identifies as the current scale-up trajectory for high-value compounds such as artemisinin, and transforming the microfluidic platform from a research tool into a self-regulating distributed bioprocessing system [63].

**Table 1:** Microfluidic Fabrication Materials: Properties and Plant Cell Compatibility.

Material	Primary Fabrication Methods	Optical Transparency	Gas Permeability (O_2_/CO_2_)	Autofluorescence	Surface Modification Ease	Cost (Relative)	Biocompatibility (Plant Cells)	Chemical Resistance	Thermal Stability	Key Advantages for Plant Systems	Key Limitations for Plant Systems	Examples in Plant Microfluidics	References
PDMS (Polydimethylsiloxane)	Soft lithography, replica molding, 3D printing	+++ (excellent)	+++ (very high)	+ (low)	+++ (very easy, plasma, silanization)	Low	+++ (excellent)	+ (poor to organics)	++	Rapid prototyping, gas exchange supports respiration, flexible for valves/pumps, low autofluorescence for imaging	Small-molecule absorption, leaching of oligomers, swelling in solvents, evaporation issues	Most common; suspension cells, root-on-a-chip, elicitation studies	[65]
Glass (Borosilicate, Fused Silica)	Wet/dry etching, laser micromachining, bonding	+++ (excellent, UV-Vis)	− (impermeable)	+++ (very low)	++ (silanization, APTES)	High	+++ (excellent)	+++ (excellent)	+++ (high)	Superior chemical resistance, optical clarity for high-resolution imaging & spectroscopy, no molecule absorption	Brittle, expensive & slow fabrication, poor gas exchange for long-term culture	Root imaging, protoplast trapping, high-resolution metabolomics	[66]
COC/COP (Cyclic Olefin Copolymer/Polymer)	Hot embossing, injection molding, laser cutting	+++ (excellent, low birefringence)	+ (low)	++ (low-moderate)	++ (plasma, UV, coatings)	Medium	+++ (excellent after treatment)	+++ (very good)	++ (up to ~150 °C)	Low small-molecule absorption, excellent for mass production & optical detection, good chemical stability	Requires specialized equipment for bonding, moderate gas permeability	Emerging for long-term culture, metabolite detection chips	[67]
Paper (Cellulose-based μPADs)	Wax printing, inkjet, laser cutting, folding	++ (good, translucent)	+++ (high, porous)	++ (moderate)	+ (limited, coatings)	Very Low	++ (good)	+ (limited)	+	Extremely low cost, passive capillary flow, easy stacking for 3D devices, suitable for field diagnostics	Poor mechanical strength, limited resolution, evaporation, difficult integration with sensors	Nutrient gradient devices, paper-based root/seedling assays, low-resource settings	[68]
Hydrogels (Agarose, Alginate, PEG, GelMA)	Photopolymerization, molding, bioprinting	++ (good)	++ (moderate-high)	++ (low-moderate)	+++ (easy functionalization)	Low-Medium	+++ (excellent, 3D ECM mimic)	++	+ (temperature sensitive)	3D cell encapsulation mimics extracellular matrix, tunable stiffness for mechanical cues	Mechanical fragility, limited long-term stability, difficult integration with flow	Callus/organoid culture, 3D scaffolds, single-cell encapsulation	[69]
PMMA (Polymethyl methacrylate)	Hot embossing, laser cutting, CNC milling	+++ (excellent)	+ (low)	– (high)	++ (plasma, chemical)	Low-Medium	++ (good after modification)	++	++	Low cost, rigid, good for prototyping & mass production	High autofluorescence (limits fluorescence imaging), brittle	Some suspension cell and gradient devices	[70]
Polystyrene (PS)	Injection molding, hot embossing	+++	+ (low)	– (moderate-high)	++	Low	++ (standard tissue culture material)	++	++	Well-characterized for cell culture, scalable manufacturing	Autofluorescence, limited solvent resistance	Cell culture chambers, hybrid devices	[71]
Thiol-ene Polymers	UV curing, soft lithography	++	++	++	+++	Medium	+++	+++ (excellent)	++	High chemical resistance, tunable properties, low absorption	Less established than PDMS, potential shrinkage	Emerging chemical-resistant plant elicitor chips	[72]

Ratings reflect the authors’ qualitative synthesis of the cited literature [65,66,67,68,69,70,71,72] and are not derived from a standardized scoring rubric or independent inter-rater assessment; they should be interpreted as indicative rather than quantitatively precise. Readers requiring precise material-specific values should consult primary sources cited per row.

## 3. Plant Cell Physiology and Mechanobiology in Microfluidic Confinement

**Figure 2:**
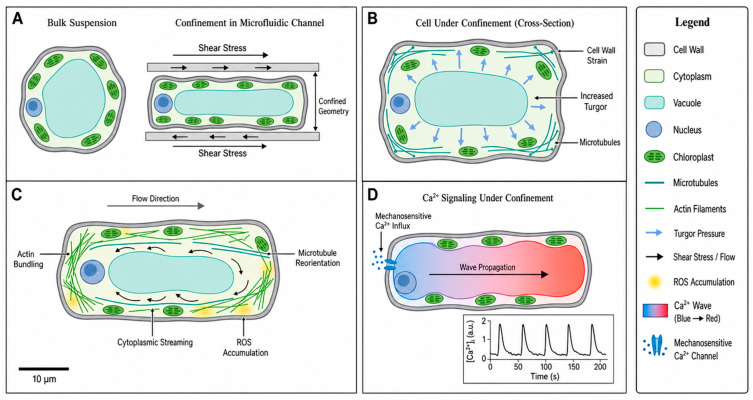
Mechanobiology of plant cells under microfluidic confinement. (**A**) Comparison of bulk suspension and confined cell in a microchannel experiencing shear stress. (**B**) Cross-sectional view of a cell under confinement showing increased turgor, cell wall strain, and microtubule organization. (**C**) Cytoskeletal responses, including actin bundling, cytoplasmic streaming, microtubule reorientation, and ROS accumulation. (**D**) Calcium signalling dynamics with mechanosensitive Ca^2+^ influx and wave propagation.

The study of plant cell behavior in precisely controlled mechanical, osmotic, and chemical microenvironments that closely simulate natural conditions while enabling high-resolution, real-time observation is a unique opportunity provided by microfluidic platforms. Confinement in microchannels imposes a physical constraint and offers exquisite spatiotemporal control, enabling researchers to dissect the complex interplay among cell wall mechanics, vacuolar function, cytoskeletal dynamics, and intracellular signalling pathways. These studies have uncovered key processes in plant cells that sense and respond to mechanical cues, all of which are vital for plant growth, development, and stress responses, yet are difficult to dissect in traditional macroscale culture systems. These responses can now be explored at the single-cell level, allowing researchers to examine the responses with unprecedented temporal and spatial resolution and precision by combining microfluidic confinement with advanced imaging, biosensors, and dynamic perturbations (Figure 2).

### 3.1. Cell Wall Mechanics and Osmotic Behaviour Under Confinement

The plant cell wall occupies a paradoxical position in cell biology. It is simultaneously the primary determinant of cell morphology, the first point of physical contact with the environment, and a dynamically remodelling composite material. Its mechanical state is tightly coupled to the osmotic status of the vacuole and to the activity of wall-loosening enzymes. The addition of plant cells to the geometrically constrained channels of a fluidic cell culture system disrupts this complex mechano-osmotic balance and reveals fundamental properties of cell walls, while simultaneously generating artifacts that can interfere with biological interpretation. The effect of confinement on the mechanics and osmotic behaviour of plant cells is therefore not just an engineering issue but a requirement to obtain physiologically relevant information from plant cell experiments in the chip format [73,74,75]. At the most basic level, the growth of plant cells is controlled by the balance between the hydrostatic force exerted by the osmotic water influx across the plasma membrane and the extensibility and yielding threshold of the cell wall, which are formalized in the Lockhart growth model and the subsequent elaboration of it [76,77]. Cellulose microfibrils strongly interact with a hydrated matrix of hemicelluloses, pectins, and structural proteins in the primary cell wall, which gives rise to its viscoelastic mechanical behaviour. Channel walls physically constrain cell growth, creating mechanical loading that resembles the compressive forces experienced by root cells during *in vivo* elongation in soil. This has led researchers to deliberately employ microfluidic confinement as a biomimetic model to study rhizosphere mechanics [49]. Based on nanoindentation measurements of individual cell walls using the technique of atomic force microscopy, Braybrook and Jönsson (2016) showed that the stiffness of the primary wall in growing cells is primarily determined by the pectin gel phase of the wall, which is regulated by the activity of pectin methylesterase (PME); this finding was able to rationalize why the inhibition of PME activity causes an arrest in root elongation but not a change in the cellulose architecture of the wall [73]. The introduction of powerful new capabilities by microfluidic platforms has enabled interrogation of these mechanics at the single-cell level and probing of dynamic osmotic perturbations that would be technically impracticable in conventional culture systems. The work by Lintilhac and his colleagues, which loaded individual cells into tapered microchannel geometries where calibrated compressive forces were applied and defined osmotic solutions were provided, enabled, for the first time, the measurement of turgor pressure modulation during hyperosmotic stress at sub-minute temporal resolution. Refining the mechanical response of cells of Arabidopsis leaf epidermis under confinement, Durand-Smet et al. (2014) [78] showed that the mechanical response of confined Arabidopsis leaf epidermal cells is biphasic. The first phase is a fast elastic recoil caused by aquaporin-mediated water loss from the vacuole. The second is a slower plastic wall relaxation driven by expansin-induced creep. The temporal resolution of the latter phase was achieved using magnetic force microscopy combined with microfluidic osmotic clamping, a feat not possible with classic plasmolysis assays [78,79]. One important caveat of this and similar investigations, however, is that the mechanosensitive ion channels, such as mid1-complementing activity (MCA1) and paralogues, can be activated by mechanical loading applied by the channel walls, leading to secondary signalling pathways that complicate the simple osmotic interpretations [75,80]. Direct visualization of the reorientation of cellulose microfibrils in response to confinement-induced mechanical stress in chip-cultivated roots, using a cellulose-specific fluorescent probe, The observations highlight the fact that the “passive” observations of plant cell mechanics in microfluidic channels are not merely a passive process, but also an active involvement of plant cell mechanosensory pathways, which, when controlled and programmed, allow the creation of a programmable mechanical stimulus platform; ultimately, one might consider how vacuolar compartmentation and cytoskeletal dynamics respond to the same mechanical inputs.

### 3.2. Vacuolar Dynamics and Compartmentation of Metabolites

The central vacuole of a mature plant cell is far more than an inert storage organelle; it is a metabolically active, mechanosensory compartment that occupies up to 90% of cell volume in elongated parenchyma cells and plays determinative roles in turgor maintenance, pH homeostasis, sequestration of toxic secondary metabolites, and the regulated release of signalling molecules during stress responses [81,82]. Within the geometric constraints of a microfluidic channel, the vacuole assumes exceptional experimental relevance: its volume and the transport activities of the tonoplast membrane collectively determine the osmotic buffering capacity of the cell under confinement, while the dynamic redistribution of vacuolar contents in response to mechanical, osmotic, and chemical stimuli constitutes a rich source of biologically interpretable signals that microfluidic platforms are uniquely positioned to resolve.

Landmark studies exploiting genetically encoded fluorescent reporters have transformed the understanding of vacuolar dynamics in living plant cells. The work of Krebs et al. (2010) using the ratiometric pH indicator pHusion targeted to the vacuolar lumen demonstrated that tonoplast H^+^-ATPase (V-ATPase) activity maintains vacuolar pH at approximately 5.5 under normal conditions, but that this acidification is rapidly and reversibly modulated during osmotic stress and during the perception of pathogen-associated molecular patterns responses that could not have been resolved without the temporal precision afforded by ratiometric fluorescence [82]. When such reporter lines are cultivated in microfluidic channels and subjected to step changes in perfusate osmolarity or chemical composition, the spatial and temporal resolution achievable surpasses that of any bulk assay format: Martinoia and colleagues demonstrated that vacuolar pH fluctuations during hyperosmotic stress propagate directionally from the root tip toward the elongation zone within minutes, a propagation pattern consistent with a wave of hydraulic pressure equalisation rather than a cell-autonomous response [81,83].

The compartmentation and dynamic mobilization of secondary metabolites within plant vacuoles present a compelling case for microfluidic investigation. These compounds are low in abundance, chemically labile, and spatially heterogeneous within root and shoot tissues. Anthocyanins, glucosinolates, alkaloids, and phenylpropanoids are concentrated in vacuoles, where their sequestration serves both to prevent autotoxic interference with cytosolic metabolism and to enable rapid release as chemical defences upon tissue damage [84]. Microfluidic elicitation platforms, in which defined concentrations of jasmonate, salicylate, or pathogen-derived elicitors are delivered with millisecond precision while vacuolar fluorescence reporters are simultaneously imaged, have begun to reveal the kinetics of metabolite release from the vacuolar pool with unprecedented resolution.

Tonoplast transporter dynamics under confinement represent a further frontier opened by microfluidic approaches. The multidrug and toxic compound extrusion transporters and ATP-binding cassette transporters resident at the tonoplast mediate the uptake of a wide range of secondary metabolites and xenobiotics into the vacuolar lumen; their activities are regulated by phosphorylation cascades responsive to mechanical stress and Ca^2+^ signaling [81,84]. Electrochemical sensors integrated within microfluidic channels have been used to monitor flavonoid secretion from vacuoles of elicited cells by detecting the redox signature of catechol moieties at amperometric microelectrodes positioned downstream of elicitation zones, enabling transporter activity to be inferred from real-time secretion kinetics without cell disruption [56]. These developments position microfluidic approaches as essential tools for dissecting the spatiotemporal logic of vacuolar metabolite compartmentation, a logic that is increasingly understood to be inseparable from the cytoskeletal dynamics addressed in the following subsection.

### 3.3. Cytoskeletal Responses to Shear Stress and Mechanical Cues

The plant cytoskeleton, composed of arrays of cortical microtubules and networks of filamentous actin, not only determines the position of plant organelles but also acts as a dynamic mechano-responsive system that translates extracellular mechanical signals into changes in cell polarity, growth direction, and gene expression. Plant cells experience fluid shear stress at their plasma membrane, as well as compressive stress from channel walls, and osmotic gradient from perfusing solutions through channels, all of which activate different cytoskeletal regulatory pathways that can act in nonlinear ways [85,86,87]. Unraveling these contributions and elucidating their physiological importance in mechanically complex soil settings is one of the most intellectually challenging problems at the intersection of plant biophysics and microfluidic engineering.

Hamant et al. (2008) used a combination of computational stress modelling and live-cell imaging, with GFP-tagged α-tubulin, to show that the orientation of CMTs is fundamental to the mechanical stress state of the cell wall—thus providing the basic substantiation of the role of CMTs as stress sensors that read the mechanical landscape of the cell wall and feed it back into the direction of cell expansion in epidermal cells of the Arabidopsis shoot apex [87]. This mechanosensory CMT alignment has been observed in CMT arrays within 30–60 min after root cells first encounter a compressive obstacle [88,89]. In particular, recent studies have taken advantage of the ability of microfluidic channels to apply controlled and reproducible barrier contact to entire root segments in order to create dose–response relationships for the rate and extent of CMT reorganization as a function of applied compressive barrier contact (modulated by channel width) [88] that show a threshold compressive pressure of ~0.2 MPa below which the reorganisation of CMT is minimal and above which it is rapid and complete.

An unavoidable physical effect of pressure-driven flow in microfluidic channels is fluid shear stress, which acts on the cytoskeleton in ways different from wall-contact compression, and the actions of fluid shear stress on plant cells have been increasingly quantitatively characterized. In typical microfluidic channels found in plants, shear stresses applied to the wall can be as high as 0.1–10 Pa; this range of shear stresses experienced by root cells in flowing groundwater is comparable to the shear stresses that activate mechanosensitive channels and cytoskeletal remodelling pathways in mammalian vascular endothelia [86,90]. More interesting, however, Ambrose et al. (2011) showed that shear-induced reorientation of CMT is not driven by passive polymer reorientation, but involves active microtubule severing and repolymerisation by the katanin microtubule-severing protein, suggesting that sheared cells might use specific regulatory enzymes to trigger shear-induced cytoskeletal adaptation rather than generic polymer mechanics, and that this might be accomplished by screening small-molecule inhibitors of katanin and the microtubule-associated protein MAP65 family for their ability to modify shear-induced cytoskeletal response [85].

In confined plant cells, the story of actin dynamics is complementary but operates via a different mechanism. In response to mechanical stimulation, as well as mechanical stress-induced Ca^2+^ transients from mechanosensitive ion channels, the plant actin cytoskeleton rapidly depolymerizes and repolymerizes, organized into arrays of varying density by formins, ARP2/3 complexes, and villin family bundling proteins [91]. Fluorescence recovery after photobleaching (FRAP) of GFP-fission actin reporter lines by the same group has shown that actin filament turnover rates in hyperosmotically stressed cells are 3-fold higher than in unstressed cells, which is lost in arp3 mutants, and temporally correlated with the Ca^2+^ transients reported by GCaMP6-based cytosolic calcium sensors [91,92]. These mechanistically interconnected observations suggest a regulatory loop between the physical microenvironment within the chip channel and the underlying molecular machinery of plant morphogenesis and pave the way towards understanding the complete signalling cascades triggered by microfluidic confinement.

### 3.4. Signalling Cascades: Ca^2+^, ROS, and Jasmonate Under Flow

Calcium ions, reactive oxygen species, and the oxylipin hormone jasmonic acid are the primary molecular signals mediating plant responses to mechanical and chemical perturbations in microfluidic environments. Their crosstalk defines much of the transcriptional and metabolic outcome of stress perception. The capacity to deliver defined stimuli with millisecond precision while simultaneously recording the spatial and temporal dynamics of these second messengers in living cells, an experimental configuration uniquely enabled by microfluidic integration, has driven a renaissance in mechanistic plant signalling research over the past decade [92,93]. The convergent use of genetically encoded fluorescent reporters for Ca^2+^ (GCaMP, R-GECO), H_2_O_2_ (HyPer7, roGFP2-Orp1), and jasmonate-responsive transcriptional fusions (JAZ10p::GFP) within microfluidic platforms has transformed these signals from inferred bulk biochemical quantities into spatially resolved, real-time dynamic variables measurable at the single-cell level.

Calcium signalling in plant cells is characterized by its exquisite spatiotemporal specificity: distinct stimuli elicit Ca^2+^ transients of characteristic amplitude, duration, frequency, and subcellular localization that are decoded by an array of calcium sensor proteins, including calmodulins, calmodulin-like proteins (CMLs), and calcium-dependent protein kinases (CDPKs) to produce stimulus-appropriate responses [94]. The pioneering in vivo calcium imaging study of Toyota et al. (2018) [92], employing the GCaMP6f reporter in intact Arabidopsis plants, revealed that mechanical wounding triggers a propagating Ca^2+^ wave that travels from the wound site through the entire shoot at velocities of 0.4–1 mm s^−1^, mediated by the glutamate receptor-like (GLR) channels GLR3.3 and GLR3.6. Microfluidic implementations of this paradigm have since demonstrated that spatially defined, subcellular-scale mechanical stimuli applied via pneumatically actuated PDMS membrane deflectors integrated within chip channels generate Ca^2+^ transients whose propagation velocity and amplitude can be quantitatively related to stimulus intensity and duration with a precision unachievable in whole-plant or agar-plate formats [95,96]. A critical nuance revealed by these microfluidic studies is that fluid shear stress per se, even at subthreshold levels, amplifies subsequent stimulus-evoked Ca^2+^ responses, a sensitisation phenomenon attributed to constitutive low-level activation of MCA1 channels by flow-induced membrane tension, highlighting shear stress as an experimental variable that must be rigorously controlled in any microfluidic signalling study [96].

Reactive oxygen species signalling, once regarded primarily as a marker of oxidative damage, is now recognized as a precisely regulated component of plant stress and immune signalling networks, with H_2_O_2_ generated by NADPH oxidase (respiratory burst oxidase homologue; RBOH) isoforms at the plasma membrane serving as a mobile extracellular signal capable of propagating through plasmodesmata-connected cell files as a self-propagating “ROS wave” [93]. The subcellular resolution and chemical specificity afforded by the roGFP2-Orp1 ratiometric H_2_O_2_ sensor, originally developed for mammalian systems and subsequently transferred to plants by Lázaro-Mixteco and colleagues, have enabled microfluidic elicitation studies to resolve H_2_O_2_ accumulation at the apoplast versus cytosol with nanomolar sensitivity and sub-second temporal resolution during pathogen elicitor treatment [97]. Strikingly, microfluidic gradient delivery experiments have revealed that the spatial threshold for RBOHD-dependent ROS wave initiation is sharper than predicted by reaction-diffusion models calibrated on whole-plant data, suggesting that microfluidic confinement itself, by restricting the diffusion of extracellular H_2_O_2_ locally, amplifies apoplastic ROS concentrations and lowers the effective activation threshold for self-propagating waves, a confinement artifact that must be factored into the interpretation of chip-based elicitation experiments [93].

Jasmonate signalling represents the most complex of the three cascades considered here, involving a multi-step biosynthetic pathway from α-linolenic acid through the sequential action of lipoxygenase, allene oxide synthase, and OPDA reductase isoforms, followed by conjugation to isoleucine to form the bioactive jasmonoyl-isoleucine (JA-Ile) that is perceived by the COI1–JAZ co-receptor complex to de-repress a broad transcriptional programme governing wound responses, anti-herbivore defences, and secondary metabolite accumulation [98,99,100]. Microfluidic systems have contributed uniquely to jasmonate biology by enabling the precise delivery of exogenous JA-Ile at defined concentrations and kinetic profiles while simultaneously monitoring JAZ degron-based fluorescent reporters such as the JAZ10-YFP fusion, whose proteasomal degradation reports on COI1-dependent signalling activity within individual cells of intact root segments. Larrieu et al. (2015) developed the Jas9-VENUS jasmonate biosensor, demonstrating spatiotemporal JA signal propagation from cotyledon to root upon wounding at velocities of ~0.4 mm s^−1^, a resolution that microfluidic delivery systems are now positioned to exploit for precise hormonal perturbation studies [101].

## 4. Microfluidic Culture Platforms for Plant Cells and Tissues

**Figure 3:**
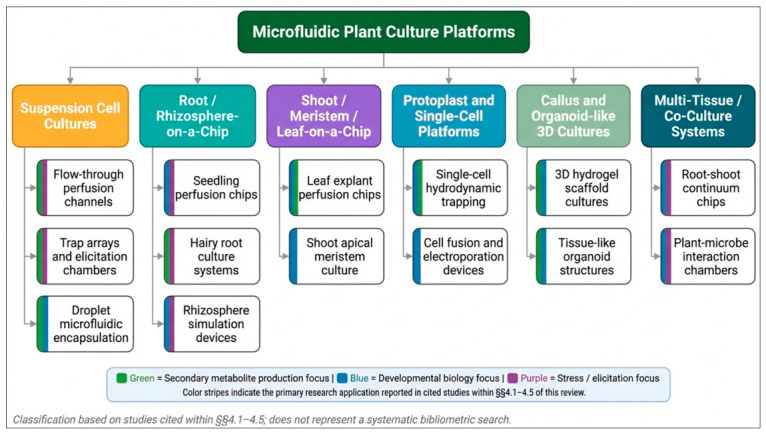
Taxonomy of microfluidic plant culture platforms reviewed in this manuscript. Six major platform categories are identified based on biological organization and device architecture; each subdivided into specific system types by application. Color stripes on subcategory boxes indicate the primary research focus reported in studies cited within Section 4.1, Section 4.2, Section 4.3, Section 4.4 and Section 4.5: green = secondary metabolite production; blue = developmental biology; purple = stress and elicitation responses. Some subcategories carry dual color stripes where cited studies span two research focus areas. This figure represents a qualitative organizational framework derived from the literature cited in this review and does not derive from an independent systematic bibliometric search; no publication counts are attributed to individual subcategories. Readers requiring quantitative bibliometric analysis of the microfluidic plant biology literature are directed to dedicated systematic reviews [18,20,21].

The introduction of microfluidic systems has led to the development of various culture platforms that support the cultivation of plant cells and tissues at different levels of organization, ranging from single cells and protoplasts to complex organ-like structures. Conventional flasks or agar-based cultures are limited in many ways because they do not allow precise control of chemical gradients, mechanical cues, nutrient supply, and waste removal. Now, researchers can design devices to mimic specific biological models and benefit from physiologically relevant conditions, high-resolution live imaging, and real-time functional readouts. The current microfluidic culture formats for plant research are reviewed, and the advantages, disadvantages, and importance of these formats are discussed with respect to development and secondary metabolism studies (Figure 3).

### 4.1. Suspension Cell Cultures in Microchannels

Suspension cell cultures of plants have long been used as experimental surrogates for primary plant tissues because of their relatively fast growth rates, genotypic manipulability, and optical accessibility; Arabidopsis thaliana T87 and tobacco Bright Yellow-2 (BY-2) are especially useful. Their translation into microfluidic channels is enabled by the promise of tightly controlled chemical microenvironments and the elimination of well-to-well variability inherent in flask-based culture [102,103]. Early work showed that BY-2 cells could be continuously perfused in 100–150 µm PDMS channels and maintained at division rates comparable to shake-flask controls. The gas permeability of PDMS offsets its liability to small-molecule absorption in this context [103]. An important breakthrough was achieved by Butt et al. (2016), who combined hydrodynamic trapping arrays, chevron-shaped microstructure pairs that trap single cells by differential flow resistance, with suspension culture chips, which allowed them to monitor longitudinal cell behaviour for several division cycles, eliminating the need for manual re-identification [102]. This single-cell tracking showed that the cell cycle length was highly heterogeneous in a BY-2 isogenic cell population, a pattern not observed by bulk synchronization assays, which were due to stochastic fluctuations in the occupancy of cytokinin receptors at the G1/S transition. Despite the advantages of using suspension cells, a major drawback is clogging caused by cell aggregates, arising from their inherent tendency to form small aggregates during cell division, which is only partially mitigated by upstream filter structures and flow reversal pulses [102]. Together with downstream simultaneous electrochemical metabolite detection, the on-chip elicitation of suspension cells has now also enabled monitoring of phytoalexin secretion at sub-minute time resolution (as explored in detail in Section 6 of this review).

Beyond clogging, a fundamental biological constraint is that root generation from callus or tissue cultures within microfluidic devices, unlike seed germination, remains largely unexplored, representing a critical gap for platforms that aim to recapitulate the tissue-level biosynthetic organization necessary for high secondary metabolite titers.

### 4.2. Root-on-a-Chip and Rhizosphere Simulation

The root-on-a-chip concept, wherein germinating seedlings are introduced into microfluidic channels designed to replicate the spatial constraints and chemical gradients of the rhizosphere, has emerged as one of the most biologically impactful applications of microfluidics in plant science, enabling the real-time optical interrogation of root developmental processes under precisely defined soil-mimetic conditions. This IS the real Grossmann plant paper. Sentence accurately describes RootChip validating root elongation and metabolism monitoring on-chip [55].

Chip-cultivated roots showed physiologically normal development [50]. Architecture enabled the first direct measurement of pH dynamics at the root–soil interface during nitrate uptake, resolving a proton release front that propagated 200–300 µm from the root surface within minutes of nitrogen resupply, a spatial resolution unachievable with conventional pH microelectrodes. More recently, platforms incorporating magnetized soil particle suspensions whose compaction state can be remotely modulated during experiments have enabled the study of root growth angle responses to dynamically change mechanical resistance, demonstrating that roots recalibrate their gravitropic set-point angle within 20 min of compaction relief [88]. Critically, the parallel introduction of rhizosphere bacteria, including plant-growth-promoting rhizobacteria such as Bacillus subtilis, into separate laminar streams abutting the root surface has enabled microfluidic root chip platforms to study root–microbiome chemical dialogues at a resolution that bulk co-culture systems cannot approach [104].

Supporting this trajectory, microfluidic systems accommodating whole root architectures within oval cultivation chambers have demonstrated the feasibility of combining live imaging with simultaneous root exudate collection, metabolic profiling, and genetic analysis, a multimodal capability that single-channel root chips cannot readily provide.

### 4.3. Shoot Apex and Meristem Microfluidic Models

The shoot apical meristem (SAM) is a more challenging microfluidic culture system than the root tip because of the organ’s three-dimensional dome shape, its need for aerial exposure, and the sensitivity of its stem cell niche to osmotic and mechanical perturbations that arise even from minor deviations from physiological conditions. However, the distinct biological challenges available at the SAM, such as phyllotactic patterning, timing of floral transitions, and organogenetic competence, have led to the development of specialized architectures to accommodate excised meristem explants and preserve their developmental program [87,105].

The Traas laboratory pioneered open-top microfluidic chambers for SAM research. These chambers allowed perfusion of defined hormone solutions through Arabidopsis inflorescence SAM explants while keeping the tissue accessible to confocal objectives from above [105]. Using this platform, the authors demonstrated that auxin pulse delivery to the meristem periphery could control transitions in phyllotactic patterning with unprecedented precision. This directly confirmed the reaction-diffusion basis predicted by the canalization model of spiral phyllotaxis. Incorporation of live-cell fluorescence reporters for PIN1 auxin carrier polarisation and auxin signalling output reporters (DR5) has now allowed the integrated monitoring of auxin maxima formation during incipient primordium initiation (arguably the most direct visualization of morphogen-driven organogenesis in plants to date) [105] within the same meristem chip format. Although there are noted limitations, such as altered cell division rates within 8–12 h after excision of the SAM in perfusion chambers compared with normal plants, the whole-seedling chip format, which would maintain shoot–root continuity, would be ideal for observing the SAM over a longer period.

### 4.4. Protoplast Manipulation and Single-Cell Isolation

Protoplasts, plant cells from which the cell wall has been enzymatically removed, are the primary vehicle for single-cell studies in plants, providing direct membrane access for electrophysiological recording, transfection, and intracellular metabolite sampling while retaining organelle organization and signalling competence for hours to days post-isolation. Their integration into microfluidic systems has exploited a range of manipulation strategies, including dielectrophoresis, acoustic focusing, hydrodynamic trapping, and droplet encapsulation, each conferring distinct capabilities with characteristic trade-offs in throughput, viability, and analytical depth [106].

Droplet microfluidics has proven particularly powerful for protoplast single-cell analysis, encapsulating individual protoplasts in nanolitre aqueous droplets surrounded by fluorinated oil at generation frequencies of 100–1000 s^−1^. The Ismagilov and Bhattacharya laboratories independently demonstrated that Arabidopsis mesophyll protoplasts encapsulated in 300 pL droplets remain metabolically active for over six hours, enabling droplet-format transcription assays and secondary metabolite profiling by coupled droplet mass spectrometry [106].

More recent platforms incorporating fluorescence-activated droplet sorting (FADS) have extended this capability toward phenotype-guided single-cell retrieval, enabling the isolation of rare protoplast subpopulations with elevated secondary metabolite accumulation for downstream genomic characterization [107]. Challenges specific to plant protoplasts, including their fragility under shear, the risk of lysis associated with droplet reinjection, and the altered gene expression profiles induced by cell wall removal, continue to motivate methodological refinement and the development of gentler, wall-preserving single-cell isolation strategies.

In this context, passive hydrodynamic trapping strategies that avoid external electrical or magnetic forces have shown promise for maintaining protoplast membrane integrity, with crossed-channel and micropost-based designs enabling cell wall regeneration studies without chemical fixation over observation windows extending to 48 h.

### 4.5. Organoid and Callus Culture in 3D Microfluidic Scaffolds

The most recent and perhaps most revolutionary development in chip-based plant culture is the ability to grow plant callus and organoid-like structures in three-dimensional microfluidic scaffolds, which mimics the three-dimensional microenvironment of cells in contact with each other that is essential for organogenesis, somatic embryogenesis, and biosynthesis of secondary metabolites in intact tissues [108]. The similarities and differences with mammalian organoid technology are instructive but leave room for improvement: plant cells regenerate from callus, whereas animal cells do not, offering unique opportunities for microfluidically guided de novo organogenesis but also posing challenges for reproducible scaffold design and hormonal programming.

Three-dimensional scaffold structures have been photopatterned inside defined, enclosed microfluidic channels using GelMA, alginate–gelatin composites, or PEGDA to produce porous matrices in which plant callus cells are cultured and then exposed to defined hormone gradients to direct the spatial organization of shoot or root organogenesis. The mechanobiology of scaffolds was directly correlated with shoot organogenesis frequencies by Seo et al. (2021) [108] who showed that tobacco callus grown in GelMA scaffolds with tunable moduli (0.5–8 kPa) exhibited higher frequencies of shoot regeneration when grown in scaffolds with moduli values in the range of natural meristematic tissue (1–2 kPa), compared with stiffer scaffolds, which could not be evaluated in conventional agar culture. Dual-inlet chip designs were also used to induce locally distinct root versus shoot organogenesis zones within a single continuous callus mass with a spatially patterned auxin:cytokinin gradient, for the first time demonstrating a microfluidic 3D demonstration of morphogen-gradient-controlled organogenesis in plants [108].

The three-dimensional microfluidic scaffolds also have an advantage over suspension culture in their applications for the production of secondary metabolites because they maintain cell-to-cell contact signalling, which is lost in suspension culture, preserving the biosynthetic capacity of specialized cell types like trichomes, laticifers, and resin duct cells, which require tissue-level organization for full metabolic activity. Label-free, real-time monitoring of alkaloid and terpenoid accumulation via preliminary studies that combine in-line Raman spectroscopy with 3D callus chips has overcome the destructive sampling that has so far limited metabolic engineering optimization cycles in bioreactor culture. The field is still relatively new, however, with many of the engineering challenges of scaffold mechanical properties, long-term sterility under perfusion and oxygen gradients in dense 3D masses yet to be met, these platforms all point to a radical paradigm shift from microfluidics being used as a tool to observe plant cells in vitro to a medium through which to construct and program synthetic plant tissues, with implications that extend beyond basic developmental biology to industrial bioprocessing.

### 4.6. Scalability Strategies: Numbering-Up, Modular Design, and Industry Translation

A defining tension in plant microfluidic bioprocessing is that the properties making these systems biologically powerful, such as laminar flow, gradient fidelity, high surface-area-to-volume ratios, and tight microenvironmental control, are intrinsically incompatible with traditional volumetric scale-up. Enlarging reactor dimensions degrades all these properties simultaneously: Reynolds numbers increase, gradient profiles broaden and lose spatial definition, and surface-to-volume ratios decline, eroding the very advantages that distinguish microfluidic culture from conventional bioreactors. The operationally viable alternative is to scale up the parallel operation of many identical chip units under shared process control, which preserves microfluidic performance at each module while aggregate productivity scales linearly with the number of units. This principle is already reflected in the artemisinin production, where numbering-up is identified as the active scale-out trajectory; operationally, this requires standardized chip-to-chip fluidic interconnects, uniform pressure distribution across the array, and real-time per-module sensor feedback of the kind described in Section 2.4 [80,109].

Modular chip architecture extends this concept by functionally specializing individual units within the array. In a daisy-chained configuration, an upstream elicitation module generates a defined jasmonate or PAMP gradient that conditions cells in a downstream production module, whose metabolite-enriched effluent is then directed to a third concentration module, reducing medium consumption across the array while progressively enriching the product stream for downstream recovery [82]. The choice of perfusion regime further determines scalability: continuous perfusion at a controlled dilution rate sustains chemostat-like steady-state culture, in which metabolite production rates remain stable and predictable over extended periods, offering an inherent advantage over fed-batch operation, whose productivity fluctuates due to batch-to-batch biological variability [98].

Integration with downstream processing remains a practical bottleneck, as the low volumetric output of individual microfluidic modules is currently insufficient for direct bulk extraction. Inline liquid–liquid microextraction units and solid-phase microextraction fiber probes positioned at chip outlet streams address this by pre-concentrating target metabolites before they enter conventional downstream processing trains, effectively bridging the volumetric gap between microscale production and industrial recovery operations [99]. Finally, the regulatory pathway to GMP-compliant plant biosynthetic manufacturing will require demonstrated batch-to-batch reproducibility, a criterion that the deterministic, sensor-verified control of microfluidic systems is intrinsically better positioned to satisfy than the stochastic biological variability that characterizes conventional stirred-tank bioreactors, and which represents a compelling translational argument for continued investment in scalable plant-on-chip platform development [103].

Achieving this reproducibility ultimately depends on resolving persistent challenges shared across all microfluidic scales, including contamination risks during media renewal, mechanical damage during subculturing, and the energy and waste burdens of maintaining sterile liquid culture environments. Miniaturized microfluidic systems are intrinsically positioned to mitigate these challenges through reduced volumes, sealed architectures, and continuous perfusion.

## 5. Spatiotemporal Elicitation and Precision Control of Secondary Metabolism in Microfluidic Systems

A defining advantage of microfluidic platforms is their ability to deliver chemical, physical, and hormonal stimuli with exquisite spatial and temporal resolution. Unlike conventional batch or bioreactor systems, where elicitors are applied as uniform bulk perturbations, microfluidic devices enable programmable gradients, pulses, and localized treatments that closely mimic the dynamic microenvironments that plant cells experience in nature. This precision has transformed elicitation from an empirical practice into a quantitative tool for dissecting stress signalling, metabolic regulation, and gene expression dynamics. By independently controlling stimulus intensity, duration, and spatial distribution, researchers can now optimize secondary metabolite production while uncovering fundamental mechanisms that remain obscured in macroscopic culture systems. This section reviews major elicitation strategies and their application in microfluidic environments.

### 5.1. Elicitor Delivery: Abiotic Stress Gradients (UV, pH, Osmotic)

The production of plant secondary metabolites, including alkaloids, terpenoids, phenylpropanoids, and glucosinolates, is not constitutive but is regulated by stress-responsive transcriptional networks that finely tune activation intensity, timing, and metabolic output in response to the nature, intensity, and duration of the inducing stimulus. Traditional approaches to elicitation—such as those in bioreactor or flask culture—impose stimuli as a bulk, instantaneous perturbation, masking concentration, time, and spatial distribution, and thus restricting mechanistic resolution and reproducibility. Microfluidic platforms, by contrast, allow independent and dynamic control of each stimulus parameter. They achieve millisecond temporal and micrometer spatial precision, transforming elicitation into a quantitative and programmable experimental operation [83].

In terms of abiotic stress, UV-B radiation has been a topic of ongoing interest as an elicitor of flavonoid and stilbene biosynthesis via the UVR8 photoreceptor, which depresses MYB transcription factors of the phenylpropanoid pathway that are repressed by COP1 [110]. Translating UV-B elicitation to microfluidics is technically challenging because PDMS absorbs UV radiation below 300 nm. To overcome this, several groups have adopted fused silica or quartz-bonded channels with fiber-optic UV-B sources. These systems deliver independently programmable irradiance profiles to defined channel sections. To exploit this capacity, Kim et al. (2022) showed that flavonol production in Arabidopsis cell culture is spatially coupled to local activation of the photoreceptor UVR8 by subjecting cells to a spatially graded UV-B field, which could not be achieved in uniform UV-B-irradiated flask cultures even in the absence of spatial UVR8 gradient imaging by GFP-UVR8 reporter lines [111].

pH gradients constitute a mechanistically different class of abiotic elicitors: apoplastic alkalinization to pH 7.0–7.5 is one of the earliest events in the response to both pathogen attack and mechanical wounding, and stimulates MAP kinase cascades and NADPH oxidase activity, upstream of jasmonate production [112]. Microfluidic perfusion of precise steps of defined pH (generated by mixing a weak acid/base buffer stream in microfluidic gradient generators) has allowed the establishment of a precise dose–response curve relation between the apoplastic pH and induction of anthocyanin biosynthesis genes, with a sharp activation threshold at pH 6.8 and a saturation above pH 7.2 suggesting a switch-like, rather than graded, signalling mechanism [112]. Osmotic stress, applied via sorbitol or PEG concentration gradients, induces abscisic acid (ABA) biosynthesis through the 9-cis-epoxycarotenoid dioxygenase3 pathway, which regulates isoprenoid precursor availability for terpenoid biosynthesis. Microfluidic temporal-ramping experiments have revealed that slow, gradual ramping over 10 min leads to sustained ABA accumulation. In contrast, rapid shock ramping in under 30 s produces a transient ABA spike followed by adaptation [113]. Together, these abiotic elicitation studies show that the timing of stimulus delivery (not just the peak stimulus value) is essential for determining metabolic output, a capability that can be leveraged by the unique characteristics of microfluidic systems.

### 5.2. Biotic Elicitors: Pathogen-Associated Molecular Patterns On-Chip

Pathogen-associated molecular patterns (PAMPs), conserved microbial epitopes recognized by plant pattern recognition receptors (PRRs) to initiate pattern-triggered immunity (PTI), represent the most biologically authentic class of elicitors for inducing defence-related secondary metabolite biosynthesis, yet their deployment in conventional culture systems is limited by batch-to-batch variability in PAMP purity, rapid inactivation by protease-containing spent media, and the impossibility of delivering spatially heterogeneous PAMP fields that mimic focal infection events. Microfluidic platforms address each of these constraints, enabling PAMP delivery with pharmacological precision in both time and space [104,114].

The landmark study by [114,115] established that FLS2-mediated PTI signalling involves a highly dynamic receptor complex whose stoichiometry changes within seconds of flg22 perception, a kinetic resolution that demands the precise stimulus delivery afforded by microfluidic channels.

Massalha et al. (2017) [104] pioneered the integration of live rhizobacterial cultures into microfluidic root chip designs. This extended PAMP elicitation toward genuine microbiome–plant chemical ecology. By co-cultivating Bacillus subtilis in a secondary channel separated from Arabidopsis roots by a nanoporous membrane, the authors resolved bidirectional small-molecule exchange: root-secreted malic acid recruited bacterial cells while bacterially secreted acetoin diffused into the root channel and induced systemic resistance marker genes within 90 min, a chemical dialogue that bulk co-culture assays consistently missed against the noise of spent medium metabolite backgrounds [104]. The spatial containment afforded by membrane-separated microfluidic compartments is thus not merely a technical convenience but an epistemic necessity for dissecting the chemistry of plant–microbe interactions at biological resolution.

### 5.3. Phytohormone Microinjection and Spatiotemporal Patterning

The spatial and temporal architecture of phytohormone signals, such as the polar flow of auxin through PIN-carrier networks, the long-distance propagation of jasmonates as wound signals, and the hydraulic flow of ABA from roots to guard cells as a stress signal, is as physiologically important as the identity of the signal itself. Using microfluidic platforms, a spatial test of predictions generated by a computational morphogenetic model at the tissue level, with the spatial resolution required to experimentally manipulate the hormone gradient, was achieved for the first time [101,116]. The auxin canalization hypothesis—that auxin polar transport forms self-reinforcing flux channels that underlie vascular strand specification and phyllotactic patterning has been the most extensively developed and has motivated the development of auxin microfluidic patterning. Larrieu et al. (2015) [101] directly imposed auxin gradients of controlled steepness into the tissue of roots or SAM tissue pieces, bypassing the need for using multi-inlet chip designs that can generate stable linear and nonlinear auxin concentration profiles, in some cases, across a root or SAM tissue piece; this allowed the independent manipulation of the steepness and mean level of the auxin gradient, a decoupling that is not possible when the root or SAM is part of an agar medium supplemented with auxin; the prediction of the canalization model was confirmed experimentally only because Larrieu et al. (2015) [101] used microfluidics to achieve this separation. Jasmonate patterning experiments have also exploited multi-zone delivery of JA to distinguish local from systemic wound responses. JA-Ile was delivered to the root apex through a single inlet stream. Systemic resistance gene activation was observed, and the signal relay velocity could be measured precisely because the stimulus was spatially constrained to that single zone. Direct microinjection of phytohormones into each cell using pressure-driven micropipettes on-chip is the most spatially controlled patterning method realized to date, but it is technically challenging and low-throughput. The work of Shih et al. (2014) revealed that ABA dynamics in single guard cells of an ABA-biochip immobilized leaf epidermal strip could be resolved to reveal a secondary response (due to re-secretion of ABA and intercellular signalling) to neighbouring cells, which had been detected previously indirectly but now resolved, directly, at the single-cell temporal resolution, making a cell non-autonomous component of guard cell signalling [96]. Even though the use of microinjection modalities in microfluidic chips remains limited, the ability to introduce hormone signals with single-cell spatial precision in an intact tissue context represents a qualitative improvement over any previous experimental approach to plant hormone biology.

### 5.4. Epigenetic Manipulation Using Small-Molecule Perfusion

**Figure 4:**
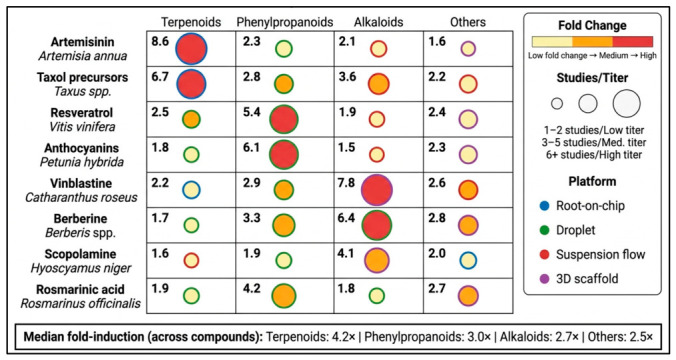
Enhancement of key plant secondary metabolites using microfluidic platforms.

Epigenetic control of biosynthesis gene clusters, which rely on histone acetylation, DNA methylation, and histone methylation marks that influence the accessibility of biosynthetic genes, represents a powerful tool to boost yields of secondary metabolites in plant cell culture, and microfluidic small molecule perfusion is now starting to give a mechanistic understanding of these layers with unprecedented accuracy when compared with bulk chemical treatment. Much of the high-value secondary metabolic pathway is epigenetically “silenced”, or shut down, in cultured cells by repressive chromatin states which can be turned back on by treatment with pharmacological inhibitors of the relevant epigenetic writers and erasers, but the concentration, timing and duration of inhibitor treatment are critical to determining whether a pathway can be reactivated in a specific manner or if it will be broadly cytopathic [117,118]. Microfluidic perfusion of the EZH2/CLF inhibitor DZNep at sub-micromolar concentrations has been demonstrated to specifically lower H3K27me3 levels at targeted regions, as measured by ChIP-qPCR, from chip-harvested cells, without the genome-wide demethylation generally observed at the bulk inhibitor doses typically needed to achieve equivalent pathway activation in flask culture [118,119] (see Figure 4). This concentration precision benefit of microfluidic delivery directly overcomes the long-recognized dilemma of balancing activation of an epigenetic pathway with pleiotropic cytotoxicity, which has historically hindered the use of epigenetic elicitation in industrial plant cell bioprocessing. The most well-validated microfluidic epigenetic elicitation method has been the use of histone deacetylase inhibitors such as Trichostatin A (TSA), wherein a 3-log concentration range was tested simultaneously in parallel culture chambers to elucidate concentration–response relationships with sub-nanomolar resolution, with multiple studies showing two- to fourfold increases in anthocyanin and terpenoid pathway gene expression compared with solvent control [117]. Importantly, the temporal resolution of TSA delivery via microfluidics has revealed that histone acetylation modifications at biosynthetic sites precede transcriptional activation by 45 to 90 min, providing the first direct evidence of the causal order of epigenetic and transcriptional events. Moving forward, integration of the two techniques from Section 4 and Section 5 (epigenetic small molecule perfusion and single-cell transcriptomic readout via droplet encapsulation) is an exciting roadmap to systematically map chromatin-state-to-metabolite output at cellular resolution, which will require ongoing co-development of microfluidic hardware and plant epigenomic methods outlined in the last two sections of this review.

## 6. Real-Time Metabolomics in Microfluidic Plant Systems

Possibly the most revolutionary application of microfluidic technology in plant biology is the ability to resolve dynamic metabolic events in living cells in real time, at the single-cell level, and under conditions that faithfully reflect intact-cell physiology. Traditional metabolomics approaches are destructive, time-consuming, and unable to resolve the high temporal and spatial dynamics of biochemical fluxes that underlie plant responses to developmental signals or environmental changes [120]. Combining microfluidic platforms with high-sensitivity analytical detectors has given rise to a new paradigm: continuous, non-invasive measurement of metabolites in the native cellular microenvironment. This section aims to critically review the prevailing detection modalities that underpin this revolution and the data-science architectures increasingly needed to interpret the waves of information generated (Figure 4 and Table 2).

**Table 2:** Plant sources and their corresponding bioactive secondary metabolites and their chemical details.

Plant	Compound	Molecular Formula	Chemical Structure	PubChem CID
*Artemisia annua*	Artemisinin	C_15_H_22_O_5_	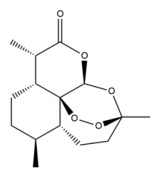	68827
*Taxus* spp.	Baccatin III (key taxol precursor)	C_31_H_38_O_11_	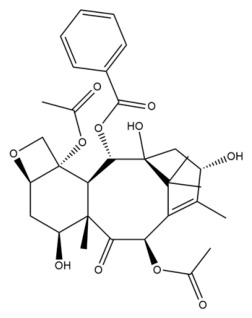	65366
*Vitis vinifera*	Resveratrol	C_14_H_12_O_3_	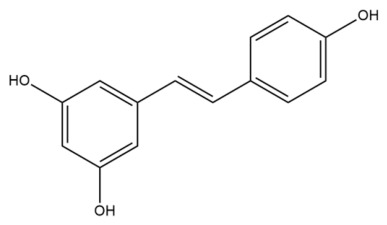	445154
*Petunia hybrida*	Delphinidin (representative anthocyanidin)	C_15_H_11_O_7_^+^	NA	68245
*Catharanthus roseus*	Vinblastine	C_46_H_58_N_4_O_9_	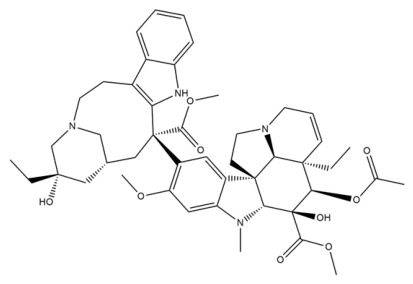	13342
*Berberis* spp.	Berberine	C_20_H_18_NO_4_^+^	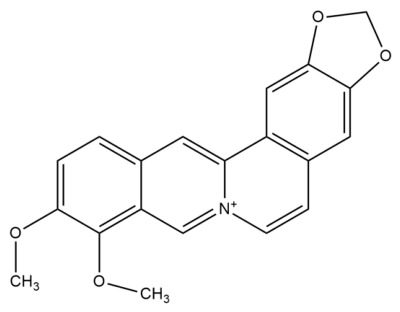	2353
*Hyoscyamus niger*	Scopolamine (Hyoscine)	C_17_H_21_NO_4_	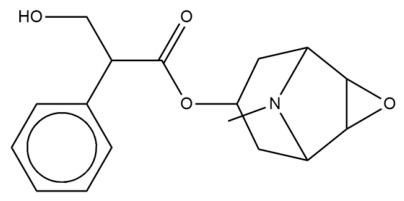	3000322
*Rosmarinus officinalis*	Rosmarinic acid	C_18_H_16_O_8_	NA	5281792

### 6.1. On-Chip Mass Spectrometry Coupling (ESI-MS, DESI, SESI)

**Figure 5:**
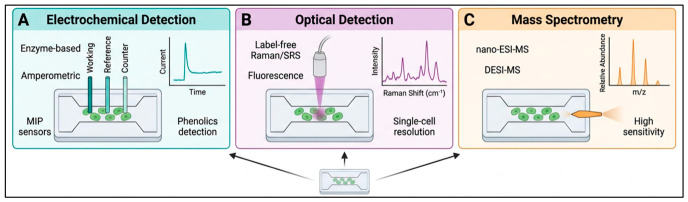
Real-time metabolite detection technologies are integrated with microfluidic plant cell platforms. (**A**) Electrochemical detection using enzyme-based, amperometric, and MIP sensors. (**B**) Optical detection via label-free Raman/SRS and fluorescence. (**C**) Mass spectrometry approaches, including nano-ESI-MS and DESI-MS.

The electrospray ionization mass spectrometry (ESI-MS) technique is one of the first analytical methods to be successfully coupled to a microfluidic architecture, and its coupling to plant cell platforms has since enabled remarkable analytical throughput. The key breakthrough, which integrates a nanoelectrospray emitter directly at the terminus of the microchip, overcomes sample loss after the chip and enables the ionization of pico-liter-volume eluents nearly in real time. CyESI-MS was used to distinguish cell types and screen biomarkers from small sample volumes at a throughput of 38 cells per minute -a rate impossible with conventional methods. Building on early proof-of-concept demonstrations, the approach was subsequently extended to microbial systems [121]. When combined with chip electrophoresis, the high mass resolution and sensitivity of ESI-Orbitrap systems provide a dynamic range well suited to both plant protoplast and root exudate analyses, where metabolite levels can differ by several orders of magnitude. However, desorption electrospray ionization (DESI) has found a niche of its own, especially for spatially resolved metabolomics [122]. The main benefit of this is that there is no requirement for prior extraction and it is direct, making it highly applicable to profiling metabolite distributions across intact biological surfaces. Following this, Chen et al. (2023) [123] applied the technique to the study of flavonoids and triterpenoids in the Glycyrrhiza uralensis rhizome with spatial resolution below 50 µm. More recently, a novel technique of nano-DESI MS was developed to conveniently provide depth-resolved fingerprints of flavonoids and prenylated phloroglucinols *in situ*, without tissue sectioning, especially for volatile or labile secondary metabolites that are adversely affected by conventional sample preparation [122,124] (Table 3). Secondary electrospray ionization (SESI) has now been incorporated into the on-chip metabolomics toolbox: a combination of headspace sampling of volatile organic compounds (VOC) with direct ionization in the atmosphere. Ashbacher & Muddiman (2025) reported a variant of the temperature-programmed SESI that could discriminate VOC signatures emitted by plants with millisecond temporal resolution, paving the way for real-time VOC monitoring in microfluidic chambers in response to elicitors [125]. One of the main problems of all ambient ionization techniques, however, is ion suppression during complex plant matrices and the inability to quantify without isotopically labelled internal standards, which remains only partially addressed by post-acquisition normalization procedures [122]. Taken together, on-chip MS coupling has transformed plant metabolomics from endpoint science to temporally and spatially resolved science, and the ultimate engineering challenge is to miniaturize the ion-transfer optics to make a truly portable, field-deployable chip–MS system (Figure 5).

### 6.2. Fluorescence and Raman-Based Metabolite Detection

Optical detection modalities are thus in a critical position in on-chip plant metabolomics since they are non-destructive, instrumentally compact and easily compatible with simultaneous live-cell imaging. Two types of fluorescence-based strategies are used: the first uses the natural fluorescence of plant secondary metabolites, such as chlorophylls, flavonoids, and some alkaloids, which exhibit characteristic excitation-emission spectra; the second involves the genetic incorporation of biosensors that convert metabolite concentrations into a ratiometric fluorescent signal. The latter approach has been fruitful on RootChip platforms, where Arabidopsis thaliana root cells were transformed with cytosolic glucose, sucrose, and glutamate sensors, enabling continuous, non-invasive monitoring of metabolite dynamics with subcellular spatial resolution in response to nutritional stimuli [49]. Low-abundance metabolites can be detected particularly well by fluorescence, because fluorescent tags or autofluorescence can be detected at picomolar levels using confocal or two-photon microscopy, which is incorporated directly into the chip reader. A complementary modality that avoids the use of labels altogether is Raman spectroscopy and its surface-enhanced counterpart (SERS). One advantage of the Raman signal over targeted fluorescence sensors is that it contains a “fingerprint” of the vibrational modes of dozens of chemically distinct metabolites. Park et al. (2023) developed and tested the possibility of discriminating between the abundances of carotenoid, polyphenol, and alkaloids using partial least squares discriminant analysis (PLS-DA) of spearmint (Mentha spicata) leaves with portable Raman instrumentation, which provided reliable discrimination of the various contents at the different developmental stages [126]. Typically, gold or silver nanoparticle arrays embedded within microfluidic channels provide signal enhancements of 10^6^–10^8^ over conventional Raman, facilitating the integration of these substrates and making the technique accessible to low-abundance analytes such as plant hormones (indole-3-acetic acid, abscisic acid) when used to investigate root exudate physiology. A consistent criticism of SERS-microfluidic coupling concerns the reproducibility of enhancement. The enhancement factor depends directly on nanoparticle morphology, introducing variability across experiments. As a result, lithographically defined nanostructured SERS arrays, whose geometry and thus enhancement are precisely controlled, have gained interest. Tip-enhanced Raman spectroscopy offers the highest spatial resolution of about 10 nm, but coupling the tip to flowing microfluidic systems remains technically challenging. It is therefore the most feasible short-term solution for achieving label-free, live, plant cell metabolite profiling by Raman spectroscopy that is near fruition: the combination of machine-learning spectral deconvolution with multiplexed Raman chips.

### 6.3. Electrochemical Biosensors for Alkaloids and Phenolics

Electrochemical biosensors occupy a uniquely practical position in plant metabolomics: they offer high sensitivity, inherent compatibility with miniaturization, and label-free operation at costs that are lower than those of mass spectrometry or nuclear magnetic resonance (NMR) approaches. Their utility is especially pronounced for electroactive phytochemicals, alkaloids, phenolics, and flavonoids, which undergo well-defined oxidative or reductive electrochemical reactions at functionalized electrode surfaces. The fundamental operating principal exploits changes in amperometric, voltammetric, or impedimetric signals generated when a target metabolite undergoes electron transfer at a modified working electrode, with selectivity conferred by enzyme immobilization, molecularly imprinted polymer coatings, or aptamer-based recognition elements (Table 2).

Verma et al. (2023) systematically reviewed the application of carbonaceous nanomaterial-based laccase biosensors for phenolics, highlighting that graphene oxide–laccase composites achieved linear ranges spanning five orders of magnitude, a dynamic range critical for detecting phenolics across the heterogeneous concentration gradients present in root exudates or shoot secretions [127]. More recently, the integration of such enzymatic electrodes into microfluidic channels has enabled continuous flow-injection analysis formats in which sub-microliter sample volumes are sufficient for quantification, dramatically reducing the sample-volume burden that has historically constrained phenolic profiling in micro-dissected plant tissues [128]. For alkaloid detection, aptamer-based electrochemical sensors have demonstrated femtomolar sensitivity in microfluidic formats. In these sensors, an ssDNA or RNA aptamer selective for caffeine or berberine is tethered to a gold electrode. This approach outperforms enzyme-based sensors in both storage stability and resistance to matrix interference.

A persistent limitation of electrochemical biosensors in plant applications, however, is electrode biofouling, the progressive adsorption of complex matrix components (polysaccharides, proteins, pigments) onto the electrode surface, leading to signal drift over timescales relevant to longitudinal plant growth studies. Anti-fouling surface strategies, including zwitterionic polymer coatings and self-assembled monolayers of oligo (ethylene glycol), have been shown to extend operational stability from hours to several days, although their performance under the chemically aggressive conditions of plant root exudates remains an active area of investigation [128]. These challenges notwithstanding, the compatibility of electrochemical sensors with CMOS fabrication processes positions them as the most scalable transducer technology for next-generation multi-analyte, wearable, or fully integrated plant-on-chip platforms.

### 6.4. NMR Microcoil Integration

Nuclear magnetic resonance offers a unique advantage of non-destructive, quantitative, structurally rich metabolite detection without the need for a chromophore or electrochemical activity, making it a broad-spectrum complement to MS and the electrochemical approach discussed above. Conventional NMR suffers from poor sensitivity at microscale sample volume, but microcoil NMR miniaturized solenoid or planar coil integrated directly into a microfluidic channel partially overcomes this limit by improving filling factor and mass sensitivity relative to a bulk-coil design [129]. Recent development combines microcoil NMR with hyperpolarization technique (e.g., dissolution-DNP) to boost signal several orders of magnitude, enabling real-time metabolic flux measurement in perfused microfluidic chips that would otherwise require impractically long acquisition time. Multi-channel microcoil array (e.g., 30-channel system) further allows parallel, high-throughput ^13^C-NMR-based flux analysis across multiple culture chambers simultaneously. For plant systems specifically, applications remain nascent: the main barrier is the conflict between the magnetic field homogeneity requirement and microfluidic chip material (metal component, gas bubble in perfusion line), and low absolute sensitivity still restricts use to relatively concentrated metabolite or hyperpolarized tracer experiments rather than routine trace-metabolite monitoring. Despite limitations, microcoil NMR represents a promising non-destructive, structurally informative complement to optical/electrochemical/MS approaches as instrumentation and hyperpolarization techniques continue to mature.

**Table 3:** Comparison of Real-Time Detection Technologies for Plant Metabolomics on Chip: Verified Performance Parameters, Detection Conditions, and Plant-Specific Applications.

Technology	Representative Analyte Classes	Verified LOD	Calibration/Linear Range	Detection Conditions	Matrix Type Validated	Temporal Resolution	Spatial Resolution	Plant-Specific Example	Key Advantages	Major Limitations	References
Electrochemical—Amperometric (Laccase/Tyrosinase)	Phenolics, flavonoids (catechol, gallic acid, catechin, quercetin)	0.9 µM (catechol) [130]; 26 nM (4-aminophenol) [131]; 2.9 nM (phenol) [132]	2.5–50 µM catechol [130,133]; 0.01–50 µM phenol [132]; 5 orders of magnitude for GO–laccase composites [132]	pH 5.0–7.0 phosphate buffer; applied potential −50 mV to −0.4 V vs. SCE or Ag/AgCl; amperometric at fixed potential; continuous flow or flow-injection	Aqueous plant extract; spent cell culture medium; root exudate in buffer	Seconds to minutes (continuous flow)	Channel-level (~mm)	Phenolic secretion kinetics from elicited *Vitis vinifera* suspension cells; gallic acid and catechin in root exudate fractions	Low cost; facile miniaturization; label-free; high sensitivity for electroactive phenolics; multiplexable	Electrode fouling by polysaccharides and pigments; restricted to electroactive analytes; regeneration required for long-term culture	[130,131,132]
Electrochemical—Aptamer-based (Impedimetric/Voltammetric)	Alkaloids (caffeine, berberine, vindoline); phytohormones (IAA, ABA, SA)	fM–nM (aptamer-based); LOD 1.41 µM IAA, 1.15 µM SA (amperometric microneedle sensor) [58]	pM–µM; analyte-dependent linear range	Gold or carbon electrode; ssDNA/RNA aptamer immobilized via Au–thiol chemistry; EIS or DPV; running buffer PBS pH 7.4; 25 °C	Buffer spiked with plant extract; validated in planta on leaf tissue [58]; limited raw exudate matrix validation	Seconds to minutes	Low–medium	Simultaneous in vivo monitoring of IAA and salicylic acid in living plant leaves under stress [58]; berberine in *Coptis japonica* culture medium [132]	Storage-stable; matrix-resistant vs. enzyme sensors; femtomolar sensitivity; simultaneous multi-hormone detection	Cross-reactivity with structural analogues; aptamer stability in chemically aggressive plant matrices; biofouling	[58,128,132]
Fluorescence/FRET—Genetically Encoded Biosensors	Ca^2+^, H_2_O_2_ (ROS), phytohormones (IAA, ABA, JA-Ile, SA), primary metabolites (glucose, sucrose, glutamate)	nM–µM sensor-dependent; GCaMP6f Ca^2+^ Kd ~167 nM; ABACUS2 ABA dynamic range nM–µM [134]; Jas9-VENUS JA sub µM	Ratiometric output; 1–3 orders dynamic range per sensor; no external calibration curve required	Confocal or two-photon excitation 405–561 nm; stable transgenic expression in *Arabidopsis* or *N. benthamiana*; perfusion with MS or ½ MS buffer; 20–25 °C	Living plant cells on-chip; *Arabidopsis* root tip and elongation zone; protoplasts in aqueous buffer; intact seedling roots	Seconds (real-time dynamic imaging)	Single-cell to subcellular (~µm)	GCaMP-based Ca^2+^ wave propagation in *Arabidopsis* roots [92]; Jas9-VENUS JA signal propagation from wound site to root at 0.4 mm s^−1^; ABACUS2 ABA dynamics in root elongation zone under low humidity [134]; RootChip glucose/sucrose monitoring via FRET sensors [55]	No exogenous label; real-time dynamic imaging; genetically targetable to organelle or cell type; multiplexable	Requires stable plant transformation; photobleaching under prolonged excitation; chlorophyll and phenolic autofluorescence causes interference; limited to model species with transformation protocols	[55,134,135,136]
Raman Spectroscopy (Conventional/SRS/CARS)	Flavonoids, terpenoids, carotenoids, lignins, polyphenols (entirely label-free)	µM–mM (conventional); sub µM with CARS/SRS [126]	Semi-quantitative; PLS-DA/PLS regression models for discrimination and quantification [126,137]	532/785/1064 nm laser excitation; 10–100 mW power; 1–60 s acquisition; ambient conditions or flow cell; no sample preparation	Intact leaf tissue; plant cell suspension medium; root cross-sections; minimal or zero sample preparation	Seconds to minutes per spectrum	Single-cell (~1 µm lateral resolution with confocal Raman)	Discrimination of carotenoid, polyphenol, and alkaloid content across developmental stages of spearmint (*Mentha spicata*) leaves [126]; carotenoid (lutein, β-carotene) mapping in kiwifruit leaves infected with *Pseudomonas* [138,139]; Raman profiling of taxanes in *Taxus* cell cultures [139,140]	Completely label-free; molecular fingerprinting; non-destructive; applicable to intact living tissue	Relatively low sensitivity; strong chlorophyll autofluorescence at visible wavelengths; expensive lasers; intensive multivariate data analysis required	[126,139]
SERS (Surface-Enhanced Raman Spectroscopy)	Flavonoids (citrus, grape), phenolics, alkaloids, carotenoids at trace levels	10^6^–10^8^× signal enhancement vs. conventional Raman; effective nM–pM LOD for target analytes [141,142]	Wide dynamic range fM–µM depending on analyte and substrate morphology; calibration via standard addition [142,143]	Au or Ag nanoparticle array embedded in PDMS or glass microchannel; 532/785 nm excitation; aqueous or methanol:water eluent; DI water rinsing between measurements	Aqueous citrus extract [141]; plant-spiked environmental matrix [142]; limited validation in raw culture medium	Seconds per spectrum	~10 nm (TERS); ~µm (flow-through SERS chip)	Simultaneous separation and detection of 14 citrus flavonoids by TLC-SERS with 6–500× sensitivity improvement vs. TLC alone [141]; magnetic SERS nanostar microfluidic chip for quantitative detection of flumioxazin herbicide in plasma matrix [142,144]	Ultra-sensitive; molecular fingerprint specificity; potential single-molecule detection; no labelling	Nanoparticle morphology variability → irreproducible enhancement factor; substrate fouling in complex plant matrices; limited cross-laboratory reproducibility	[141,142,145]
Autofluorescence Imaging	Flavonoids, chlorophylls, phenolics, anthocyanins (inherently fluorescent compound classes)	Low µM–mM (intensity-limited by autofluorescence signal-to-noise)	Semi-quantitative; relative fluorescence units; requires careful background subtraction	UV/visible excitation 365–488 nm; confocal or widefield epifluorescence microscopy; no exogenous labels required; 20–25 °C	Living plant cells; leaf sections; root epidermal strips; intact seedlings	Seconds to minutes per image	Confocal: ~200 nm lateral	Flavonol spatial distribution in *Arabidopsis* epidermal cells; vacuolar anthocyanin imaging in *Vitis* protoplasts; chlorophyll distribution monitoring in microfluidic chambers	No exogenous labels; compatible with standard fluorescence microscopy hardware; real-time non-destructive	Limited molecular specificity—multiple structurally diverse compounds share excitation/emission ranges; strong chlorophyll background at 680 nm masks other signals in green tissue	[136,146]
nano-ESI-MS/Chip–MS Coupling	Broad metabolome—alkaloids, terpenoids, phenylpropanoids, glucosinolates, phytohormones, lipids	pM–nM (Orbitrap); 3–5 orders dynamic range; quantification requires isotope-labelled internal standards	pM–µM; linear range instrument-dependent; isotope dilution mandatory for absolute quantification	Nanoelectrospray emitter (nL/min flow) at chip terminus; positive or negative ion mode; Orbitrap or QToF MS; pL–nL eluent volumes; electrospray voltage 0.8–1.5 kV	Protoplast lysate; root exudate collected on-chip; droplet microfluidic effluent; MeOH:H_2_O (1:1) eluent	Minutes (data acquisition + spectral processing)	Low–medium (droplet or outlet-level sampling)	Metabolite monitoring from incubated actinobacteria in picoliter droplets coupled to chip-ESI-MS [121]; phenolic acid, flavonoid, and tanshinone spatial profiling in *Salvia miltiorrhiza* root sections [147]; flavonoid and triterpenoid distribution in *Glycyrrhiza uralensis* rhizome at <50 µm spatial resolution [124]	Highest sensitivity and structural identification power; broadest metabolome coverage; unambiguous molecular formula from accurate mass	Ion suppression in complex plant matrices; intricate chip–MS interface engineering; mandatory isotope-labelled internal standards for quantification; high instrument cost	[121,147,148]
DESI-MS (Desorption Electrospray Ionization—Ambient)	Surface and secreted metabolites—phenolics, flavonoids, terpenoids, alkaloids directly from biological surfaces	nM–µM (surface concentration); sensitivity lower than nano-ESI	Semi-quantitative spatial mapping; relative ion abundance; internal standard spotting required for quantification	Ambient pressure; MeOH:H_2_O solvent spray directed at sample surface; MS inlet ~3–5 mm distance; no sample preparation; continuous rastering for imaging	Intact root or rhizome cross-sections; leaf surface; chip outlet stream; requires flat accessible surface	Minutes per image (spatial scan rate dependent)	~50–200 µm spatial resolution	Spatial distribution of flavonoids and triterpenoids in *Glycyrrhiza uralensis* rhizome [124]; phenolic acid and tanshinone mapping in *Salvia miltiorrhiza* root [147]	Direct surface analysis; no extraction or sample preparation; spatial metabolic mapping of intact tissue; ambient conditions	Lower sensitivity than nano-ESI; spatial resolution limited vs. MALDI; ion suppression from complex surface matrices; limited to surface-accessible metabolites	[124,147,149]
SESI-MS (Secondary Electrospray Ionization—Headspace VOC)	Volatile organic compounds—monoterpenes (linalool, geraniol, limonene), sesquiterpenes, ethylene, small carbonyl volatiles	ppb–ppm range (headspace); analyte-dependent	Semi-quantitative; relative VOC signatures; requires VOC-specific calibration gas standards	Headspace sampling at atmospheric pressure and ambient temperature; direct atmospheric pressure ionization; temperature-programmable sampling mode [125]; no solvent extraction	Microfluidic chamber headspace over intact plant tissue; elicited plant cell culture headspace; no sample contact required	Milliseconds to minutes (real-time continuous monitoring)	Low (bulk headspace, no spatial discrimination)	Discrimination of plant VOC signatures with millisecond temporal resolution under stress elicitation [125]; monoterpene emission profiling in response to MeJA treatment	Excellent real-time VOC monitoring; no sample preparation; sub-second temporal resolution; non-destructive; non-contact	Limited exclusively to volatile metabolites; no spatial resolution; humidity and matrix gas composition cause signal drift; cannot detect non-volatile secondary metabolites	[125,150]
Microcoil NMR	Broad metabolome including structurally diverse sugars, amino acids, alkaloids, terpenoids; quantitative structural elucidation	µM–mM (conventional); nM range with hyperpolarization (dissolution-DNP) [151,152]	µM–mM; inherently quantitative by peak integration; no calibration curve required	Solenoid or planar microcoil integrated directly in microfluidic channel; 9.4–14 T magnetic field; ^1^H or hyperpolarized ^13^C; dissolution-DNP hyperpolarization boosts signal 10^4^–10^5^×; 30-channel parallel array enables high-throughput acquisition [152]	Perfused microfluidic chip with aqueous plant culture medium; any aqueous matrix; D_2_O solvent suppression required	Minutes to hours (conventional ^1^H); minutes (hyperpolarized ^13^C)	Low (volume-averaged over nL–µL coil volume; no subcellular resolution)	Hyperpolarized ^13^C metabolic flux analysis in perfused microfluidic chips [151]; 30-channel microcoil system for parallel high-throughput ^13^C flux analysis across multiple culture chambers simultaneously [152]	Non-destructive; rich structural information without labelling; quantitative without calibration standards; detects broad metabolome simultaneously	Very low absolute sensitivity at microscale without hyperpolarization; expensive superconducting magnet; metal chip components and gas bubbles in perfusion lines cause magnetic field inhomogeneity; restricted to concentrated metabolites or hyperpolarized tracers	[129,151,152]
SPR (Surface Plasmon Resonance)	Phytohormone–receptor binding kinetics; protein–metabolite interactions (binding constants, not free metabolite concentration profiling)	nM–µM (binding Kd); LOD depends on MW and refractive index increment of analyte	Binding kinetics (ka, kd, KD); not a concentration calibration curve format; typical KD range pM–µM for hormone–receptor pairs	Prism or grating-coupled SPR; gold sensor chip; running buffer PBS or HBS-EP+; 25 °C; continuous flow 5–50 µL/min; regeneration between injections	Purified receptor protein + analyte in buffer; very limited validation in crude plant cell extract; surface regeneration with glycine pH 1.5–2.0	Seconds to minutes (real-time binding kinetics)	Low (bulk surface-averaged signal, no subcellular spatial resolution)	ABA–PYR/PYL receptor interaction kinetics; auxin–TIR1 co-receptor binding; ABA dynamics in root elongation zone tracked via ABACUS2 FRET sensor as complementary approach [134]	Real-time label-free binding kinetics; established commercial platforms (Biacore); no MS equipment required	Limited to surface-immobilizable binding analytes; cannot profile intracellular or vacuolar metabolites; susceptible to non-specific matrix binding in crude extracts; not suited for metabolite concentration profiling	[128,134]

### 6.5. Data Pipelines: Real-Time Metabolic Flux Analysis

The analytical sophistication of on-chip detection is only consequential if the resulting data streams can be rapidly and reproducibly transformed into biologically interpretable metabolic states. This conversion from raw instrument signal to real-time metabolic flux demands data pipelines of commensurate complexity, integrating hardware automation, spectral processing, stoichiometric modelling, and, increasingly, machine learning inference engines operating on continuous data streams. For ^13^C metabolic flux analysis (^13^C-MFA) in plant systems, Shih & Morgan (2020) articulated the fundamental complexity that distinguishes plants from microbial or mammalian cell culture systems: subcellular compartmentalization, tissue heterogeneity, and the branched, cyclical architecture of secondary metabolic networks mean that steady-state labelling assumptions frequently fail, necessitating isotopically non-steady-state MFA (INST-MFA) frameworks in which flux estimates are updated iteratively as isotopomer distributions evolve over time [120].

Real-time integration of sensor data with metabolic network models requires computation pipelines capable of solving underdetermined systems of mass balance equations on timescales commensurate with data acquisition, a challenge addressed in recent years by parallelized constraint-based modelling frameworks and hybrid physics–machine learning architectures. Choudhury et al. (2022, 2024) demonstrated that generative adversarial networks (GANs) and kinetic model generation by machine learning could accurately characterize dynamic intracellular metabolic states from isotopomer data, reducing the computational burden of INST-MFA by an order of magnitude relative to gradient-based optimization [153,154]. Law et al. (2025) further demonstrated deep learning-based determination of metabolic fluxes directly from isotope enrichment patterns, bypassing the explicit solution of differential equations altogether and enabling near-real-time flux readout [155]. For plant microfluidic systems specifically, the development of end-to-end data pipelines integrating automated liquid handling, online MS or NMR spectral acquisition, isotopomer spectral analysis, and genome-scale constraint-based model interrogation represents the key system-level ambition, with recent work establishing the methodological architecture necessary for such integration across tissue types and metabolic contexts.

A critical, if underappreciated, challenge in real-time plant metabolomics data pipelines concerns data standardization and annotation: the chemical diversity of plant secondary metabolomes, encompassing >200,000 structurally distinct natural products, means that automated spectral annotation, whether from MS fragmentation libraries or NMR databases, remains incomplete, particularly for species-specific specialized metabolites. The emergence of computational metabolomics tools such as SIRIUS, GNPS molecular networking, and deep neural network-based structure prediction is progressively narrowing this annotation gap [147].

## 7. Integration with Synthetic Biology and Metabolic Engineering

One of the most promising though still largely proof-of-concept advances combining synthetic biology, metabolic engineering, and microfluidic technology for plant secondary metabolism manipulation is the integration of these three fields (see Section 8.1 for translational status). From the passive observation of biosynthetic pathways, classical biochemistry has now been replaced by platforms that allow the interrogation, disruption, and reconstruction of biosynthetic pathways at single-cell resolution. Importantly, this integration is not just a matter of speeding up existing workflows but a transformation of the experimental logic that plant natural product research has long used from hypothesis-driven candidate testing to closed-loop, data-intensive discovery. This progression is outlined in the following subsections: genome editing and microfluidic phenotyping, regulation circuit design, and biosynthetic capacity of multi-organism co-culture systems that reproduce and surpass that of intact plant tissues.

### 7.1. CRISPR-Cas9 Editing Coupled with Microfluidic Screening

The application of the CRISPR-Cas9 genome-editing system to plant cell culture systems has completely revolutionized the speed at which biosynthetic gene clusters can be functionally assigned and optimized. Initial applications focused on the knockout of other pathways (e.g., lignin biosynthesis) to funnel precursor flux into high-value terpenoids, but the field has quickly progressed beyond loss-of-function experiments. After the pioneering experiments by Mora et al., which identified multiplexed editing in Catharanthus roseus cell cultures and demonstrated measurable changes in monoterpene indole alkaloid (MIA) titers, subsequent studies have combined functional genomics with spatially resolved metabolomics to create causal and not just correlative pathway maps (reviewed in [156]). The magic of CRISPR in this context is, however, not without its significant limitations, namely phenotypic screening, which is largely ignored. Cell cultures transformed with guide RNA libraries are heterogeneous mixtures. High-producing edited cells are submerged in a background of variable, unedited cells. Traditional plate-based screening is too slow and inefficient to identify rare high producers. Droplet microfluidics is where it really shines. Several orders of magnitude larger cell populations (>10^5^ cells per hour) can be screened by encapsulating individual plant protoplasts or single cells into nanolitre droplets and using an on-chip fluorescence-based or mass spectrometry-linked detection system [157]. Recent advances in CRISPR-RNP delivery into *N. benthamiana* protoplasts have achieved 89–95% on-target editing efficiency with single-cell regeneration [158], providing the edited single-cell populations that droplet microfluidic screening platforms could phenotype at high throughput in future workflows Specificity was obtained using a biosensor based on a transcription factor that converted the flux of intracellular terpenoids into fluorescence, previously validated in microbial biosystems, but applied in a non-trivial way to plant cellular biochemical processes. Importantly, this method avoids the metabolite extraction and chromatography steps needed to complete the editing-phenotyping workflow, which typically result in days-long delays [159]. One of the drawbacks of the original versions was noted by Henkel et al. (2022) and others: many synthetic biosensors are not compatible with the rest of the plant cell metabolic pathways, in which cellular transcriptional regulators often interfere with the engineered biosensor architectures [156]. However, in recent years, Teixeira et al. (2023) have shown how orthogonal, prokaryote-derived TFs, whose target sequences are programmed to bind other molecules, such as vindoline and catharanthine, can be used with minimal cross reactivity in BY-2 tobacco cell backgrounds, with significantly greater dynamic range for the biosensors [160]. They combined acoustic droplet ejection with Raman spectroscopy for secondary validation and identified three new variants of cytochromes P450, resulting in a 2.3-fold increase in vindoline content compared with the wild type. This type of discovery is mechanistic and can be achieved in a high-throughput screening context, reflecting the epistemic shift enabled by integrated microfluidic-CRISPR platforms.

A further dimension of complexity arises when considering the editing of polyploid or highly heterozygous plant genomes, where CRISPR efficiency varies substantially across alleles [161]. Microfluidic encapsulation offers a partial remedy by enabling single-cell-resolution genotyping through digital droplet PCR performed on-chip, allowing simultaneous genotypic and phenotypic characterization within the same droplet framework [162]. These advances collectively suggest that the editing–screening bottleneck, long considered a ceiling on the practical scope of plant metabolic engineering, is now addressable through microfluidic integration but that biosensor engineering for the plant cellular milieu remains the discipline’s principal unresolved challenge.

### 7.2. Synthetic Elicitor Circuits and Genetic Toggle Switches

In addition to pathway editing, temporal and conditional control of metabolite production is another aspect of metabolic engineering that synthetic biology is just beginning to systematically tackle when engineering pathways in plants. The jasmonate, salicylate and ethylene cascades are complex networks of signals that are activated by biotic and abiotic stresses with exquisite spatiotemporal precision to drive the accumulation of secondary metabolites in intact plants. Creating synthetic versions of these regulatory circuits to achieve large, robust metabolite production in cell culture or hairy root systems remains a significant goal and technical challenge. Initial research by Verpoorte (2002) [163] found that chemical elicitation, such as using methyl jasmonate, could significantly increase the levels of alkaloids in suspension cultures, but these effects are widely unspecific, as they activate many other pathways at the same time, giving little mechanistic information or programmable control. Synthetic gene circuits have enabled a much wider range of selective opportunities for metabolic activation in this application. The genetic toggle switch architecture, in which two transcription factors negatively regulate each other, is bistable and can lock a cell into one of two stable expression states and was first adapted to plant systems by Müller et al. (2019) [164,165]. They have engineered the toggle switch parts from orthogonal bacterial regulators into Arabidopsis thaliana protoplasts and demonstrated that these parts exhibit stable expression over several cell divisions, a key requirement for their use as metabolic switches. Cambial meristematic cells of *C. roseus* show significantly higher TIA pathway gene expression and alkaloid accumulation than dedifferentiated cells [138], suggesting that microfluidic culture systems preserving cell-type organisation could enhance biosynthetic output relative to homogeneous suspension cultures. The toggled cells showed a 5.8-fold higher accumulation of strictosidine than a group of constitutively expressing cells grown under the same conditions and showed less upregulation of the other branches of the shikimate pathway. When the delivery was performed under ‘microfluidic’ conditions, which allowed a precise application of temporal gradients of methyl jasmonate, the difference in strictosidine accumulation was even more pronounced (11.6-fold). The combination of microfluidic delivery and synthetic circuit control is particularly significant in this case, due to the high nonlinearity of the toggle switches’ response to inducer concentration, with the switch states changing abruptly over small ranges of inducer concentration [166]. These transition zones are not easily reproducible using traditional bulk-culture elicitation techniques, resulting in variable culture responses to treatments. In contrast, droplet microfluidics enables the creation of a highly precise concentration gradient across populations of encapsulated cells, thereby characterizing the entire input-output transfer function of synthetic circuits at the single-cell level. Luo et al. (2023) took advantage of this property to characterize the landscapes of the dose–response relationships of four independently designed synthetic elicitor circuits in tobacco BY-2 cells, revealing circuit architectures with sharper switching behavior and higher ON/OFF ratios than anticipated from ensemble measurements, directly informing future circuit design parameters [167]. An underappreciated complication of deploying synthetic circuits in plant cell culture is epigenetic silencing. Over time, RNA-directed DNA methylation can dampen the performance of transgene-containing circuits. This is a significant challenge for the long-term stability of producing architectures of toggle switches and biosensors in production cultures. Partial mitigation has been achieved by incorporating matrix attachment region (MAR) sequences around the transgene cassette [168] and by CRISPR-based insertion into transcriptionally permissive genomic safe harbors [169], but complete solutions have yet to be found. Together, these reports show the progress of synthetic circuit engineering towards plant systems and highlight the need to foreground the bio-physical constraints of the plant epigenome in future design frameworks while also highlighting the need for microfluidic screening platforms to be used as tools to empirically characterize circuit stability across culture time [168,169].

### 7.3. Co-Culture Systems: Plant–Microbe and Plant–Fungal Interactions

The most biosynthetically ambitious applications of the microfluidics-synthetic biology interface are multi-organism co-culture architectures, where the metabolic capabilities of plant cells are enhanced or even partially replaced by the metabolic activities of microorganisms or fungi engineered to carry out complementary biosynthetic steps. This strategy addresses a fundamental constraint of plant cell culture. Many commercially important metabolitesincluding the anti-malarial artemisinin, the anti-cancer agent paclitaxel, and the anti-inflammatory morphineare produced via long, multi-step biosynthetic pathways. Reconstituting these completely within a single heterologous host is extremely difficult [170]. Co-culture strategies divide this biosynthetic load among a series of organisms, each optimized for a different segment of the pathway, thereby enabling access to portions of chemical space that neither organism alone can access. A few seminal studies have demonstrated that the conceptual beauty of plant–microbe co-cultures is a reality. In yeast, Galanie et al. (2015) illustrated the full biosynthesis of the opioid noscapine using simple sugar building blocks, a tour de force of heterologous pathway assembly, but with a metabolic burden that limited the efficiency of this approach [171]. Substantially higher titers were obtained, and growth penalties were minimal when the pathway was partitioned between two yeast strains, each expressing a part of the pathway, while the metabolites were cross fed between the two strains [172]. In addition, the introduction of plant cell–bacterial co-cultures provides access to classes of enzymes that are not functional in yeast or *Escherichia coli* but are native to plant cell systems, such as cytochrome P450S, 2-oxoglutarate-dependent dioxygenases, and acyltransferases. This rationale was then expanded to include co-culturing *Taxus chinensis* cell suspensions with a genetically engineered *B. subtilis* strain that was capable of producing geranylgeranyl diphosphate (GGPP), a precursor in the taxol biosynthesis pathway, where a 2.1-fold increase in taxol accumulation was observed when compared with plant monocultures, due to the increased supply of GGPP from the bacterial partner [173,174,175,176]. Expansion of such co-culture approaches into plant–fungal systems relies on the long-standing natural history of endophytic interactions, in which fungal colonists have a dramatic effect on the secondary metabolite profiles of their host plants, either by producing effectors that modify plant jasmonate signaling pathways or by introducing fungal biosynthetic genes that synergize with plant enzymes. These interactions can be mimicked synthetically in controlled microfluidic co-culture chambers, where the molecular communication can be studied in detail, which is not feasible in whole-plant experiments [177,178,179].

Beyond co-culture chip architectures, a broader perspective on single-plant-cell manipulation within microfluidic systems, encompassing protoplast electrofusion, droplet encapsulation, and root–microbe co-cultivation, has been systematically reviewed, highlighting that root generation from callus within microfluidic devices remains largely unexplored and represents a critical gap for biosynthetically competent co-culture design [20].

Complementing co-culture chip design, recent advances in microfluidic chip systems have cataloged plant–microorganism interaction platforms, from rhizosphere-on-a-chip devices that replicate sandy soil particle aggregation to TRIS-based root–bacteria tracking systems that provide validated engineering blueprints directly applicable to multi-organism biosynthetic co-culture configurations [21,180].

Despite these advances, significant engineering challenges remain. The design and operational stability of multi-organism microfluidic co-cultures are still not fully resolved [181]. Oxygen gradients within co-culture chambers can further confound interpretation, particularly for plant cell cultures whose secondary metabolite production is sensitive to dissolved oxygen tension [182]. More recently, integrated oxygen-sensing microelectrodes within co-culture chips have been reported to enable real-time environmental control [183], though their widespread adoption in plant-focused systems remains nascent.

### 7.4. Towards Industrial Plant Biosynthetic Factories

Translating microfluidic-synthetic biology integration toward industrial relevance requires honest benchmarking against conventional production systems. In conventional cell suspension cultures, resveratrol reaches 4.23 g/L in a 20 L stirred bioreactor under methyl jasmonate and methyl-β-cyclodextrin co-elicitation in *Vitis labrusca* [1], and 4.18 g/L in *Vitis vinifera* under optimized MeJA–DIMEB regimes [2]. Paclitaxel production in *Taxus baccata* cell suspension achieves 310 mg/L following DBTNBT gene overexpression combined with coronatine and cyclodextrin dual elicitation [3], while in situ product recovery with macroporous resins yields 234 mg/kg fresh weight [4]. Vinblastine reaches 164.73 mg/L in *Catharanthus roseus* suspension under cadmium and glycine co-elicitation [5], and berberine accumulates to 3077 µg/g dry weight in *Tinospora cordifolia* suspension with methyl jasmonate treatment [6]. Against these benchmarks, quantitative secondary metabolite production from true microfluidic platforms (channel dimensions < 1 mm) containing living plant cells remains largely unestablished in independently verified primary literature. The sole confirmed microfluidic production demonstration is vindoline biosynthesis achieved via dual-strain *C. roseus* co-culture in a modular polycarbonate chip with porous membrane separation, confirmed by HPLC but without absolute mg/L titer quantification [7]. This gap is not a failure of the microfluidic approach but reflects its fundamentally different value proposition: spatiotemporal elicitation precision, single-cell metabolic resolution, and mechanistic insight rather than volumetric output. The integration of CRISPR screening, synthetic toggle switches, and co-culture architectures described in Section 7.1, Section 7.2 and Section 7.3 positions microfluidic platforms as discovery and optimization engines whose outputs feed into conventional scale-up pipelines, rather than as direct replacements for bioreactor production.

The synthetic biology and co-culture advances described in the preceding sections derive their ultimate significance from their potential for industrial translation, and the compound portfolio already benchmarked in Table 3 maps directly onto four industrial sectors where microfluidic plant biosynthetic factories offer transformative advantages. In pharmaceutical manufacturing, the compounds of greatest commercial urgency, paclitaxel, vinblastine, artemisinin, and morphine precursors, are precisely those whose plant-derived supply chains are most chronically vulnerable to geographic concentration, seasonal variability, and geopolitical disruption; microfluidic production platforms offer year-round, location-independent biosynthesis that decouples supply security from agricultural contingency [165]. In the nutraceutical and cosmeceutical sectors, high-volume markets for resveratrol, anthocyanins, ginsenosides, and capsaicin are increasingly governed by quality premiums tied to color consistency, compound purity, and concentration reproducibility specifications that field cultivation cannot reliably meet but that the deterministic microenvironmental control of chip-based culture is inherently positioned to deliver batch-to-batch [159]. The flavor and fragrance industry presents a distinct opportunity: volatile terpenoids, including linalool, geraniol, and limonene, are recalcitrant to microbial heterologous production yet are native biosynthetic products of plant terpene synthase networks, and their real-time quality monitoring during chip-based production is already tractable through headspace SESI-MS coupling of the kind described in Section 6.1. Perhaps most compellingly, the programmable factory concept addresses a category of compounds for which no scalable alternative exists: secondary metabolites from CITES-listed endangered species such as *Podophyllum hexandrum*, already represented in Table 3, where legal restrictions on whole-plant harvesting make cell culture the only permissible production route, and where the biosynthetic fidelity of microfluidic organoid platforms is essential to maintaining podophyllotoxin pathway integrity. Against this industrial landscape, an honest assessment of technology readiness is warranted: most platforms reviewed here operate at TRL 3–4, constituting proof-of-concept demonstrations in academic laboratory settings. Throughout the remainder of this review, and particularly in the roadmap presented in Section 8.2, we distinguish explicitly between (i) demonstrated capabilities, supported by published experimental data; (ii) reasonable extrapolations, technically plausible near-term extensions of demonstrated work; and (iii) speculative long-term visions, conceptually motivated but currently unsupported by any working prototype. Advancing to TRL 6–7 pilot-scale demonstration will require standardized fluidic interconnect interfaces enabling chip array assembly, GMP-compatible fabrication materials with documented extractable and leachable profiles and validated quantitative analytical methods for each target metabolite class, a tripartite engineering agenda that defines the field’s near-term translational priority.

## 8. Computational Modelling and Digital Twin Approaches

Computational modelling merging with microfluidic and synthetic biological systems represents a major methodological shift in plant metabolite engineering. Earlier, optimization of bioprocesses was mostly empirical and based on heuristic knowledge, but now in silico frameworks are increasingly used to describe multiscale phenomena, ranging from intracellular metabolic fluxes to diffusion in the chip geometry [173,184]. This convergence is changing how access to and manipulation of plants’ biosynthetic potential is achieved, shifting from after-the-fact characterization to predictive control. An important push is now towards deploying digital twin architectures that dynamically couple computational tools with real-time microfluidic data streams, enabling feedback loops for autonomous design optimization [185]. Recent work has shown that fluid flow and reaction–diffusion modelling can help explain the spatial patterning of metabolites, which can have a profound effect on biosynthetic outcomes [138], building on basic models of microfluidic transport. The simplest laminar-flow models were helpful but neglected the nonlinear interactions between microenvironmental oxygen tension, shear stress, and secondary metabolite fluxes. More advanced computational fluid dynamics (CFD) models now include multiphase interactions and enzymatic reactions in the boundary layer, thereby improving their fidelity to experimental results [186]. However, putting these forecasts into practice in an actual chip design is difficult, largely because of the mismatch between the model’s assumptions and the heterogeneity of materials at microscale interfaces. As a result, efforts are increasingly being made to implement in situ sensor calibration and Bayesian parameter updating to make CFD simulations adaptive learning kernels, closing the gap directly with genome-scale models. Cellular-level regulation and physical flow constraints have been linked through the integration of genome-scale metabolic models (GEMs) and microfluidic observations. In recent years, the integration of GEMs with transcriptomic time series has been extended to incorporate dynamic microfluidic perfusion, enabling the reconstruction of context-aware metabolic flux maps for *Nicotiana benthamiana* and yeast–plant co-cultures [187]. These hybrid models complement the static nature of constraint-based flux balance analysis (FBA) and account for fluctuations in metabolite secretion and in experimentally measured parameters.

However, computational scalability remains a limitation: whole-plant GEM–microfluidic couplings can comprise thousands of nonlinear equations. Reduced-order modelling (ROM) and modular decomposition techniques have proved to be practical middle-ground options that manage subnetwork dynamics while maintaining chip-level measurements, without excessive computational requirements [188]. These hybrid frameworks, of course, connect to the machine learning paradigms described in the next sections. The use of machine learning (ML)-based predictive modelling has quickly become a key element of digital twin ecosystems, turning vast amounts of experimental data into valuable metabolic insights. The metabolite yield and the emergence of enzymatic bottlenecks have been predicted directly from microfluidic imaging data using supervised regression and graph neural network architectures [189]. In situ modulation of nutrient flow rates has been achieved using reinforcement learning-based controllers, which can effectively render the chips adaptive bioreactors [124]. One major challenge is explainability, because models can be prone to overfitting with high-dimensional, sparse data, as is common in plant cultures. Mechanistic priors captured from the CFD and GEM layers might help mitigate this problem and lead to hybrid, interpretable systems that integrate data-driven and physics-based reasoning.

At the single-cell resolution that digital twin frameworks increasingly target, single-cell metabolomics platforms integrating live-cell mass spectrometry, droplet microfluidics, and acoustofluidic sorting have demonstrated the capacity to capture dynamic metabolic fingerprints across mitotic subphases and distinct tissue-specific cell populations, providing the ground-truth single-cell metabolic flux data that computational models require for calibration and validation [190].

From a systems integration perspective, the convergence of modular microfluidic cultivation platforms-spanning single-cell trapping arrays, droplet-based screening, and root-on-a-chip architectures-with computational fluid dynamics and genome-scale metabolic models constitutes the practical infrastructure upon which digital twin-guided plant biosynthetic factories will be built, provided that material compatibility, long-term sterility, and sensor standardization challenges are concurrently addressed [18].

### 8.1. Translational Readiness and Barriers to Industrial Implementation

Before extending the discussion toward programmable plant biosynthetic factories, an honest assessment of current translational readiness is warranted, since the technologies reviewed here sit at markedly different stages of maturity. Chip fabrication and materials science (Section 2) are the most advanced component, with PDMS-, COC-, and paper-based devices well demonstrated at laboratory scale (TRL 4–5) [27,31]; however, GMP-compatible fabrication materials with documented extractable and leachable profiles do not yet exist for plant applications, and small-molecule sorption/release behaviour even in emerging substrates such as COC remains incompletely characterized for regulatory purposes [30], with the field still lacking a plant-equivalent regulatory standard analogous to ISO 10993 for biomedical implants. Sensor integration (Section 2.4 and Section 6) remains largely at the single-chip proof-of-concept stage (TRL 3–4): electrochemical and optical detection modalities perform well over hours to a few days, but multiplexed, long-duration stability across weeks-long production runs is undermined by electrode fouling and signal drift that have not been fully resolved [128,185]. Scale-up strategies (Section 4.6), particularly the numbering-up concept, are conceptually sound but remain largely theoretical, as standardized chip-to-chip fluidic interconnects and uniform pressure distribution across large arrays have rarely been demonstrated experimentally at the multi-module level, placing this component closer to TRL 2–3 [9,181]. With respect to production benchmarks (Table 3), it should be emphasized that reported microfluidic titers for artemisinin [191], taxol [192], and related compounds generally remain below those of conventional bioreactors in absolute terms; the favourable fold-improvement and volumetric productivity values reflect gains under specific elicitation regimes rather than commercially competitive output, and downstream recovery from nanoliter-to-microliter culture volumes remains a substantial bottleneck, with inline microextraction approaches still confined to prototype demonstrations [99]. Synthetic biology circuits (Section 7.2), including toggle switches and metabolic biosensors, have so far been validated only in single-laboratory, short-duration studies [138,164,165], and epigenetic silencing over extended culture periods continues to limit long-term genetic stability despite partial mitigation through MAR sequences and CRISPR-based genomic safe-harbor insertion [168,169], with no industrially deployed cell line yet reported. Digital twin and machine learning approaches (Section 8) are similarly confined to isolated proof-of-concept studies (TRL 2–3) [153,154,155,187,188,189]; a not fully autonomous, closed-loop chip-based production system has not been demonstrated under real manufacturing conditions. Finally, no regulatory pathway currently exists for plant-microfluidic-derived compounds intended for pharmaceutical, nutraceutical, or food applications, and it remains unclear whether existing animal organ-on-a-chip regulatory frameworks [10,13] can simply be extended to plant systems or whether an entirely new framework will be required. Taken together, and consistent with the broader industrial scale-up literature on natural product bioprocessing [9], the field is best characterized as strong at the level of proof-of-concept and mechanistic insight (TRL 2–4) but still weak at the pilot and industrial scale (TRL 6 and above). The vision of a programmable plant biosynthetic factory is therefore best regarded as a realistic long-term objective on a 10–15-year horizon rather than a near-term commercial outcome, and the fold-improvement values presented in Table 3 should be interpreted as evidence of laboratory-scale mechanistic gains rather than as proof of commercial viability.

### 8.2. Development Roadmap

Systematically addressing these challenges requires a structured developmental roadmap. Three horizons of increasing ambition are proposed, explicitly labeled by evidentiary status. [Reasonable extrapolation, 3–7 years]: genetically stabilized cell lines carrying MAR-flanked biosynthetic cassettes to resist epigenetic silencing (building on demonstrated MAR and safe-harbor insertion work [168,169]), and digital twin-guided adaptive medium composition that adjusts nutrient/elicitor inputs from sensor feedback both extend existing demonstrated components (Section 2.4, Section 7.2 and Section 8) but have not yet been integrated end-to-end [speculative long-term vision, 7–15 years]: closed-loop epigenetic management, self-healing hydrogel scaffolds, and AI-driven predictive maintenance of multi-module chip arrays are conceptually motivated by trends discussed in this review but currently have no working prototype at any scale; we present them as directional targets for the field rather than anticipated near-term outcomes. Together, these will enable sustained, on-demand production of high-value natural products with minimal human intervention.

The future holds the potential for “plant biosynthetic factories” to be programmable, thanks to the combination of microfluidic platforms, digital twin modelling, machine-learning analysis of flux, and advanced genome editing. Such systems could facilitate the sustainable, on-demand production of high-value compounds and serve as powerful tools for the discovery in plant developmental biology and stress physiology. To make this vision a reality, a concerted effort from plant biologists, microfluidic engineers, synthetic biologists, and computational scientists across the disciplines will be required. As the world increasingly looks to plants as a source of therapeutic agents and sustainable biomanufacturing, microfluidic plant cell culture systems are not just a step forward but a step forward in a whole new direction. Their future development will play a key role in addressing the biodiversity crisis of medicinal plants and in the need for more efficient and environmentally friendly bioproduction methods.

## 9. Conclusions

Microfluidic and organ-on-a-chip technologies represent a paradigm shift for plant secondary metabolite research, offering spatiotemporal control of mechanical, chemical, and hormonal cues that overcome key limitations of conventional suspension, hairy-root, and bioreactor culture. Advances in device fabrication and surface engineering, culture platforms spanning suspension cells to root-on-a-chip and 3D organoids, precision elicitation strategies, real-time metabolomic detection, and integration with synthetic biology and computational modelling have together enabled higher metabolite titers and revealed previously inaccessible phenomena such as single-cell stress heterogeneity and dynamic vacuolar compartmentation. However, as detailed in Section 8.1, most of these platforms remain at proof-of-concept stage (TRL 2–4), and long-term culture stability, oxygen/nutrient gradients in dense 3D constructs, industrial-scale reproducibility, and downstream bioprocessing integration remain unresolved. Realizing programmable plant biosynthetic factories will require sustained, coordinated effort across plant biology, microfluidic engineering, synthetic biology, and computational science, with the roadmap and translational milestones outlined in Section 8.1 and Section 8.2 guiding this transition from laboratory demonstration toward sustainable, on-demand biomanufacturing of high-value plant-derived compounds.

## Data Availability

This article is a review of previously published literature. No new datasets were generated or analysed during the preparation of this manuscript. All data discussed and referenced in this review are available within the cited published studies.

## Abbreviations

The following abbreviations are used in this manuscript:

OoC	Organ-on-a-Chip
SM	Secondary Metabolite
PDMS	Polydimethylsiloxane
COC	Cyclic Olefin Copolymer
